# Large-scale comparative analysis of cytogenetic markers across Lepidoptera

**DOI:** 10.1038/s41598-021-91665-7

**Published:** 2021-06-09

**Authors:** Irena Provazníková, Martina Hejníčková, Sander Visser, Martina Dalíková, Leonela Z. Carabajal Paladino, Magda Zrzavá, Anna Voleníková, František Marec, Petr Nguyen

**Affiliations:** 1 Faculty of Science, University of South Bohemia, České Budějovice, Czech Republic; 2 Institute of Entomology, Biology Centre CAS, České Budějovice, Czech Republic; 3 Present Address: European Molecular Biology Laboratory, Heidelberg, Germany; 4 Groningen Institute for Evolutionary Life Sciences, University of Groningen, Groningen, The Netherlands; 5 The Pirbright Institute, Surrey, UK

**Keywords:** Cytogenetics, Evolutionary biology, Genetic markers

## Abstract

Fluorescence in situ hybridization (FISH) allows identification of particular chromosomes and their rearrangements. Using FISH with signal enhancement via antibody amplification and enzymatically catalysed reporter deposition, we evaluated applicability of universal cytogenetic markers, namely 18S and 5S rDNA genes, U1 and U2 snRNA genes, and histone H3 genes, in the study of the karyotype evolution in moths and butterflies. Major rDNA underwent rather erratic evolution, which does not always reflect chromosomal changes. In contrast, the hybridization pattern of histone H3 genes was well conserved, reflecting the stable organisation of lepidopteran genomes. Unlike 5S rDNA and U1 and U2 snRNA genes which we failed to detect, except for 5S rDNA in a few representatives of early diverging lepidopteran lineages. To explain the negative FISH results, we used quantitative PCR and Southern hybridization to estimate the copy number and organization of the studied genes in selected species. The results suggested that their detection was hampered by long spacers between the genes and/or their scattered distribution. Our results question homology of 5S rDNA and U1 and U2 snRNA loci in comparative studies. We recommend the use of histone H3 in studies of karyotype evolution.

## Introduction

Cytogenetic studies aim at characterization of genome organization and its changes. Previously indispensable for the identification of genes of interest, cytogenetics may seem to struggle in the post-genomic era as it lags behind the resolution of molecular biology and genomics. Yet it remains crucial for genomic research. Cytogenetic data such as genome size and chromosome number allow for an informed choice of sequencing strategies and provide hypothetical framework for genomic studies, context to bioinformatic analyses, and physical evidence for results produced in silico^1–3^. Recent efforts, such as the Earth BioGenome project that aspire to characterize genomes of all eukaryotic biodiversity^4^, will without a doubt lead to further cytogenetic research. As a result, the new field integrating cytogenetics and genomics has recently been proposed under the term chromosomics (coined by Claussen^5^ but repurposed later by Graphodatsky^6^ and Deakin et al.^3^).

There are several approaches to distinguish individual chromosomes within a karyotype. Classical techniques such as orcein or Giemsa staining as well as various banding methods can produce chromosome-specific patterns. These techniques work very well in mammals including humans^7,8^, other vertebrates^9^, some invertebrate taxa^10–12^ and plants^13,14^. However, classical staining and banding techniques have failed in some organisms, such as moths and butterflies^15,16^.

Lepidoptera with more than 160,000 described species and great ecological diversity^17^ represent an excellent model system to study karyotype evolution and the role of changes in genome architecture in evolutionary processes. In Lepidoptera, chromosomal rearrangements such as inversions, fusions, and fissions play an important role in speciation^18^ and adaptation, such as resistance to insecticides^19,20^ and baculoviruses^21^ and detoxification of plant secondary metabolites^22^ and xenobiotics^23^. However, comparative cytogenetic studies are scarce in Lepidoptera due to the many peculiarities of lepidopteran chromosomes. Mitotic complements of both Lepidoptera and their sister group Trichoptera typically consist of a high number of small and morphologically uniform holokinetic chromosomes^24,25^. Since they lack a primary constriction, i.e. the centromere, its position cannot be used in chromosome identification^26^. Thus, cytogenetic analyses of lepidopteran karyotypes were challenging for years before molecular cytogenetic tools were introduced^25,27^ and applied on meiotic pachytene chromosomes rather than mitotic chromosomes^28^. However, broader comparative cytogenetic studies, which would help us to understand major trends in karyotype evolution of moths and butterflies, are few^29,30^ due to a lack of appropriate cytogenetic markers that can be used on this scale.

Mapping specific sequences on chromosome preparations by means of fluorescence in situ hybridization (FISH) allows us to identify particular chromosomes, study their potential rearrangements and origin, and their behaviour during cell divisions^29,31,32^. Various tandemly arrayed genes have been established as suitable markers for cytogenetic comparative studies. These universal markers have proved to be useful in a wide range of non-model species due to their conserved nature and ease of visualization by FISH methods^33–35^. The most commonly used markers are genes for major ribosomal RNAs (rDNA). Genes for 18S, 5.8S, and 28S ribosomal RNA form a transcription unit organized in clusters, which can contain hundreds or thousands of copies^36,37^. Genes for the 5S ribosomal RNA are also used^38–41^. 5S ribosomal RNA is distributed independently from the major rDNA array and is used as an independent marker. As with the major rDNA array, 5S rDNA can be localized in clusters containing tens to thousands of copies^38,42,43^ but can also be found as singular copies scattered throughout the genome^43^. Abundant data on the number and localization of both 5S and the major rDNA gene clusters in animals and plants are available in public databases^44,45^. Finally, another group of markers used in cytogenetic studies includes the uridine-rich small nuclear RNA (U-rich snRNA) genes, which are an important part of the spliceosome. For cytogenetic purposes, U1 and U2 snRNA genes have been used. U1 snRNA gene clusters have been mapped in only a few species of Orthoptera^42,46^, Isopoda^47^, and fish^48^. U2 snRNA has been used only in a few fish species (e.g. Refs.^49–51^). U1 and U2 snRNA genes are relatively new markers often used in combination with other markers as major rDNA.

Despite their easy visualization and universality, rDNA and snRNA markers also have some limitations. Their evolution is highly dynamic, and changes in their distribution do not always reflect chromosome rearrangements^46,52^. They have been compared with mobile elements and in several cases have actually been found to be associated with transposons^48,53,54^. FISH experiments using 18S and 28S rDNA genes as probes successfully revealed concealed karyotype variation between populations and closely related species of both plants (e.g. Ref.^55^) and animals (e.g. Refs.^56,57^). Therefore, rDNA and snRNA genes might be good markers for chromosome evolution between closely related species or even intra-species evolution but are less informative with increasing evolutionary scale. To study such large-scale chromosome evolution patterns, additional markers should be developed that evolve less erratically.

Despite their great potential, histone genes have rarely been used in cytogenetic studies. Histone genes encode H1, H2A, H2B, H3, and H4 proteins, which have a strong affinity for DNA. Together, the histone proteins and DNA form a nucleosome, the basic unit of chromatin^58^. Histone genes usually form tandem arrays, as this facilitates efficient transcription^58,59^. The histone genes are conserved in their protein sequence and also in the distribution of their clusters in the genome^60,61^. This makes them ideal chromosomal markers^62^ as differences in their number and position genuinely reflect chromosomal rearrangements^60^. Some examples of successful application of histone genes to fish^61^, Bivalvia^63^, and insects^34,39^, including Lepidoptera^64,65^, show their applicability in various organisms.

In this study, we analysed the chromosomal distribution of several universal cytogenetic markers, namely 18S and 5S rDNAs, U1 and U2 snRNA genes, and histone H3 genes, in 29 species of Lepidoptera to evaluate their applicability and resolution in the study of karyotype evolution. We found that some of the markers can be used successfully in all species, while others cannot be detected in certain species. To determine the reason for the unsuccessful detection of markers by FISH, we used quantitative PCR and Southern hybridization to estimate copy numbers and distribution patterns in different species. The obtained results provide not only information on the use of various markers in Lepidoptera, but also on trends in changes in the architecture of lepidopteran genomes.

## Results

### Localization of 18S rDNA and histone H3 genes

To visualize clusters of the major rRNA genes, we used FISH with a partial sequence of 18S rDNA from the codling moth, *Cydia* *pomonella* (Tortricidae) as a probe^25^. Since the nucleotide sequence of 18S rDNA is highly conserved, the probe successfully hybridized onto chromosomal preparations of all studied species sampled across the order Lepidoptera, as well as the representative of their sister order Trichoptera. Major rDNA clusters were detected at a terminal position in 22 out of 30 species. Only 6 species with interstitial clusters were documented. Multiple, up to 11, clusters were observed in approximately half of the studied species.

Although histone genes are known for their highly conserved protein sequence, they can differ significantly at the nucleotide level due to the degeneracy of codons. To ensure optimal hybridization, a fragment of the histone H3 gene was amplified, sequenced, and used as a specific probe from each species studied (Supplementary Table S1) except for few (for details see “Materials and methods”). To increase sensitivity of the FISH detection, we employed TSA-FISH which can detect unique sequences > 1300 bp^27^. In total, we successfully mapped the distribution of histone gene clusters in all studied species. In the vast majority, a single cluster was detected, located interstitially or terminally. Multiple histone clusters (2–3) were observed only in two lepidopteran species, *Tuta* *absoluta* and *Hyalophora* *cecropia*, and in the outgroup species *Glyphotaelius* *pellucidus* (Trichoptera).

**Figure 1:**
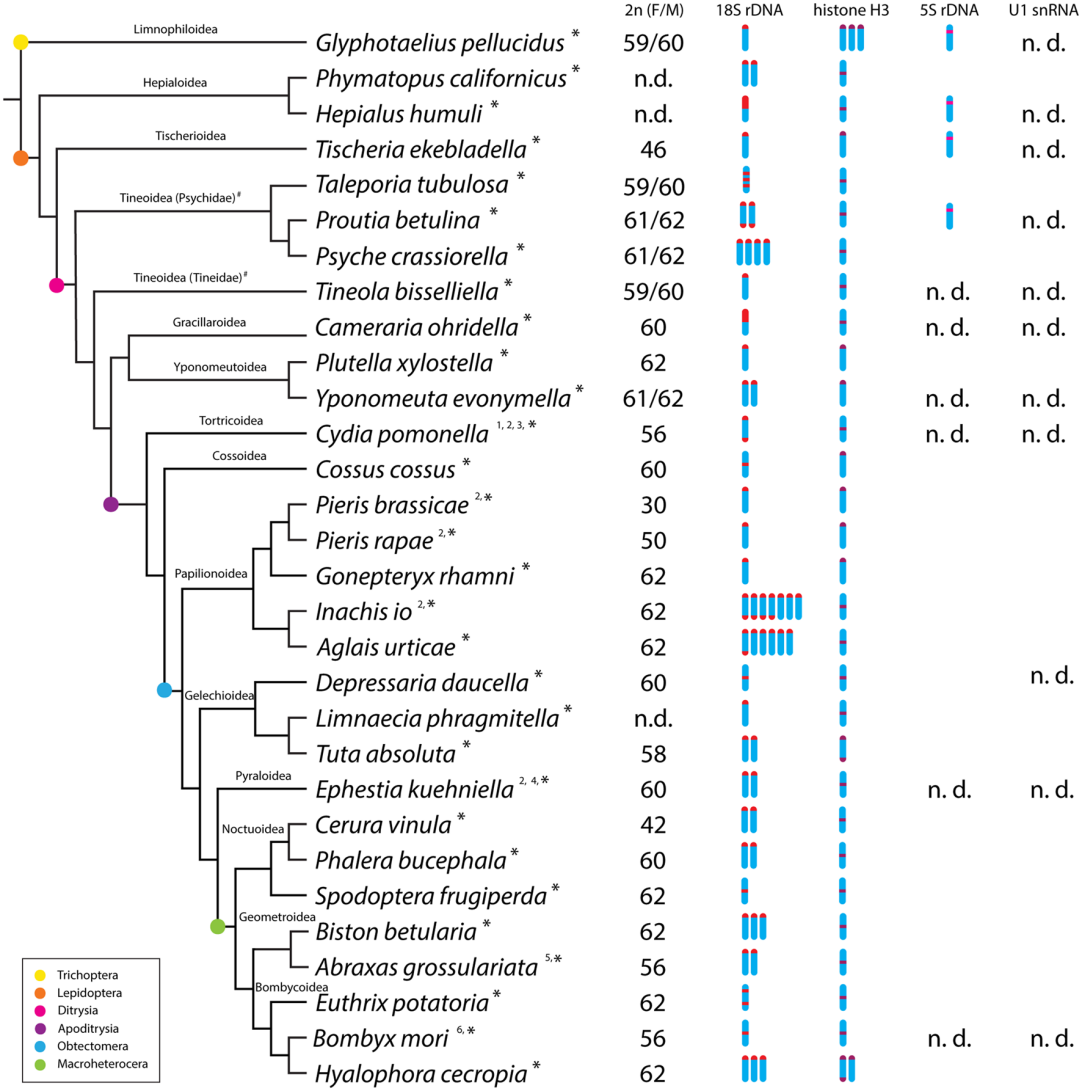
Overview of the number and position of 18S rDNA, histone H3, 5S rDNA, and U1 snRNA markers in haploid genomes of studied species. Phylogenetic relationships are based on Refs.^66–68^. ^#^Tineoidea are considered paraphyletic. Data was obtained: *in this study; 1—^25^, 18S rDNA; 2—^29^, 18S rDNA; 3—^64^ histone H3; 4—^69^, 18S rDNA; 5—^70^, 18S rDNA; 6—^31^, 18S rDNA. n.d.—not detected. F/M—female and male diploid chromosome numbers, if different. A complete list of all species analysed so far for the distribution of studied markers, including their chromosomal numbers and references, is given in Supplementary Table S2. The figure was created in Adobe Illustrator 2020, version 24.0 (www.adobe.com).

All results from the mapping of 18S rDNA and histone H3 genes are summarized in Fig. 1 and Supplementary Table S2. For a complete overview we also added information on chromosome numbers and the distribution of 18S rDNA and histone H3 genes available to date in other Lepidoptera.

### Trichoptera and non-Ditrysia

The diploid chromosome number of the caddis fly, *Glyphotaelius* *pellucidus* (Limnephiloidea) 2n♀ = 59, Z0/2n♂ = 60, ZZ, was described previously^71^. FISH experiments using 18S rDNA probe revealed a pair of terminal signals on one autosomal bivalent in this species (Supplementary Fig. S1a). Interestingly, various strong heterochromatin blocks in almost all chromosome bivalents were visible after staining with DAPI. Some of these heterochromatin patterns could potentially be used for chromosome identification. Hybridization of histone H3 probe revealed three terminal clusters of histone genes on three different bivalents (Supplementary Fig. S1b). This is one of three cases in our study where we observed multiple histone clusters (Fig. 1).

Two species of the superfamily Hepialoidea were examined, namely the ghost moth, *Hepialus* *humuli*, and the lupine ghost moth, *Phymatopus* *californicus*. Diploid chromosome numbers of these two species have not been described yet. Due to the lack of mitotic nuclei, we were not able to determine chromosomal numbers in this study. In *H.* *humuli*, the 18S rDNA probe highlighted approximately half of one pachytene bivalent (Supplementary Fig. S1c). Hybridization signals colocalized with a DAPI-positive heterochromatin block. In *P.* *californicus,* two chromosomal bivalents were detected, each bearing an rDNA cluster at the chromosome terminus (Supplementary Fig. S1e). The histone H3 probe revealed one bivalent with an interstitial cluster of histone genes in both *H.* *humuli* and *P.* *californicus* (Supplementary Fig. S1d, f).

The oak leaf miner, *Tischeria* *ekebladella,* was examined as a representative of the Tischeroidea superfamily. Its diploid chromosomal number 2n = 46 (for both sexes) was determined previously^72^. Indeed, n = 23 was confirmed in this study (Supplementary Fig. S2a). After DAPI staining, a strong heterochromatin block with terminal or subterminal location was visible in three pachytene bivalents. The subterminal heterochromatin block was adjacent to a terminal rDNA cluster highlighted by the 18S rDNA probe on one of the longer bivalents in male pachytene nuclei (Supplementary Fig. S1g). A single histone gene cluster was localized at the end of another bivalent (Supplementary Fig. S1h).

### Basal Ditrysia

Three bagworm species from the family Psychidae, namely *Taleporia* *tubulosa* (2n♀ = 59, Z0/2n♂ = 60, ZZ)*,* *Proutia* *betulina* (2n♀ = 61, Z0/2n♂ = 62, ZZ), and *Psyche* *crassiorella* (2n♀ = 61, Z0/2n♂ = 62, ZZ)^73–75^ and one species from the family Tineidae, the common clothes moth *Tineola* *bisselliella* (2n♀ = 59, Z0/2n♂ = 60, ZZ)^76^, were studied. The 18S rDNA probe revealed a distinct hybridization pattern in each species. In pachytene nuclei of *T.* *tubulosa,* an extraordinary pattern of three strong interstitial rDNA signals located on a single bivalent with regular spacing was observed (Supplementary Fig. S3a). In pachytene nuclei of *P.* *betulina,* one bivalent with signals on both ends and two bivalents bearing one terminal signal each were observed (Supplementary Fig. S3c). In *P.* *crassiorella*, four terminal signals were located on four bivalents (Supplementary Fig. S3e). In *T.* *bisselliella* male pachytene nuclei*,* a single rDNA locus was detected in a subterminal region of a pachytene bivalent (Supplementary Fig. S3g). Only one interstitial cluster of histone genes was observed in all four species, *T.* *tubulosa*, *P.* *betulina*, *P.* *crassiorella*, and *T.* *bisselliella* (Supplementary Fig. S3b,d,f,h).

The diploid chromosome number 2n = 60 of the horse-chestnut leaf miner, *Cameraria* *ohridella* (Gracillarioidea), was determined previously^77^. The 18S rDNA probe hybridized to a terminal region of one bivalent in male pachytene nuclei (Supplementary Fig. S4a). A strong, yet discontinuous signal covered approximately one fourth of the bivalent in a pattern similar to the one observed in *H.* *humuli* (see above). Mapping of the histone H3 gene showed one interstitial histone cluster on the rDNA bearing bivalent (Supplementary Fig. S4b).

Two species from the superfamily Yponomeutoidea were examined, i.e. the diamondback moth *Plutella* *xylostella* (Plutellidae; 2n = 62^78^) and the bird-cherry ermine moth *Yponomeuta* *evonymella* (Yponomeutidae; 2n♀ = 61, Z_1_Z_2_W/2n♂ = 62, Z_1_Z_1_Z_2_Z_2_; Ref.^79^ and references therein). FISH experiments carried out on pachytene nuclei of *P.* *xylostella* revealed a single terminal cluster of rDNA genes (Supplementary Fig. S4c). The histone H3 probe revealed one terminal cluster which colocalized with a strong heterochromatic block (Supplementary Fig. S4d). On chromosomal preparations of *Y.* *evonymella*, the 18S rDNA probe showed two bivalents with terminal signals of similar size (Supplementary Fig. S4e). One terminal histone cluster was observed in pachytene nuclei of *Y.* *evonymella* (Supplementary Fig. S4f).

### Apoditrysia

In a representative of the Cossoidea superfamily, the goat moth *Cossus* *cossus*, we determined the diploid male chromosome number 2n = 60 (Supplementary Fig. S5a). FISH with the 18S rDNA probe on male pachytene nuclei revealed one chromosomal pair bearing an interstitial cluster which colocalized with a small block of DAPI-positive heterochromatin (Supplementary Fig. S5a). The histone H3 probe labelled one cluster at the end of one chromosome bivalent (Supplementary Fig. S5b). However, it should be noted that due to the lack of material, our FISH experiments were performed on only one male *C.* *cossus* larva. The karyotype of the codling moth, *Cydia* *pomonella* (Totricidae), was already described as 2n = 56 by Ref.^80^ and later verified by Ref.^25^. Our results of mapping of the 18S rDNA and histone H3 probes (Supplementary Fig. S5c,d) confirmed previously published data, i.e. two rDNA clusters at both ends of a single chromosome bivalent and another bivalent bearing one interstitial histone cluster^25,64^.

### Obtectomera

Within the superfamily Papilionoidea we studied three species belonging to the family Pieridae and two species of the Nymphalidae family. The three studied pierids, namely the small cabbage white *Pieris* *rapae* (2n = 50^81^), the cabbage white *Pieris* *brassicae* (2n = 30^81^), and the common brimstone *Gonepteryx* *rhamni* (2n = 62^81^), differ in chromosomal numbers, however, mapping of the 18S rDNA and histone H3 genes revealed common hybridization patterns for both markers (Supplementary Fig. S6). Consistent with previous reports^29^, we identified one bivalent bearing a terminal rDNA cluster in *P.* *rapae* (Supplementary Fig. S6a) and *P.* *brassicae* (Supplementary Fig. S6c). We also observed this pattern in autosome pair of *G.* *rhamni* (Supplementary Fig. S6e). In pachytene nuclei of *P.* *brassicae*, small DAPI-positive blocks of heterochromatin were observed at the ends of several bivalents (Supplementary Fig. S6c). In *G.* *rhamni,* only one block of heterochromatin was visible, which colocalized with the 18S rDNA signal (Supplementary Fig. S6e). The histone H3 probe highlighted the terminal region in one chromosome pair in all three species (Supplementary Fig. S6b,d,f). Moreover, histone bearing chromosomes clearly correspond to autosomes in *P* *brassicae*, in which the sex chromosome bivalent was identified by a typical pairing of W and Z chromosomes (Supplementary Fig. S6d).

From the family Nymphalidae, the small tortoiseshell *Aglais* *urticae* and the peacock butterfly *Inachis* *io* were examined. Both species have a chromosome number 2n = 62, reported previously^81^ and confirmed in this study (Supplementary Fig. S2b,c). In male pachytene nuclei of *A.* *urticae*, six to seven small rDNA clusters were observed (Supplementary Fig. S7a). FISH with the histone H3 probe revealed one interstitial cluster colocalizing with a heterochromatin block (Supplementary Fig. S7b). Mapping of 18S rDNA genes in *I.* *io*, which was done previously^29^, revealed up to 11 small terminal clusters in pachytene nuclei, three bivalents bearing one terminal signal and four bivalents carrying terminal signals at both ends. To increase the sensitivity of detection, we repeated this experiment using TSA-FISH. Our data confirm the previous identification and distribution of eleven 18S rDNA clusters in *I.* *io* (Supplementary Fig. S7c). Similar to *A.* *urticae*, we mapped a single histone cluster to an interstitial region of a bivalent, which colocalized with a block of heterochromatin (Supplementary Fig. S7d).

We studied three species from the superfamily Gelechioidea, namely the dingy flat body moth, *Depressaria* *daucella* (Depressariidae), the shy cosmet moth, *Limnaecia* *phragmitella* (Cosmopterigidae), and the tomato leafminer, *Tuta* *absoluta* (Gelechiidae). The diploid chromosome number of 2n = 60 in *D.* *daucella* was reported recently^22^. The 18S rDNA probe revealed one interstitial cluster of major rDNA (Supplementary Fig. S8a). Similarly, we detected one interstitial histone cluster (Supplementary Fig. S8b). In *L.* *phragmitella*, the number of chromosomes was not determined previously, and we also failed to determine it due to the lack of mitotic chromosomes. However, using FISH mapping on pachytene chromosomes, we successfully identified one terminal rDNA cluster colocalized with a DAPI-positive block of heterochromatin (Supplementary Fig. S8c) and one interstitial cluster of histone genes (Supplementary Fig. S8d). In the *T.* *absoluta* strain used in this study, a diploid chromosome number of 2n = 58 was described previously^82^ and confirmed in another study^22^. The 18S rDNA probe highlighted two clusters in terminal regions of two autosomal bivalents (Supplementary Fig. S8e). Histone gene clusters were detected at both ends of a pachytene bivalent (Supplementary Fig. S8f), which makes *T.* *absoluta* one of only two lepidopteran species with multiple histone gene clusters described so far.

The only representative of the superfamily Pyraloidea included in our study was the Mediterranean flour moth, *Ephestia* *kuehniella*. Its diploid chromosome number of 2n = 60 was described previously^83^. Two terminal rDNA clusters present on two chromosome bivalents were identified by Ref.^69^, which was later confirmed by means of FISH^29^. To complete the dataset, we additionally mapped the histone H3 probe on male pachytene nuclei, which revealed one chromosome bivalent bearing a single interstitial cluster of histone genes colocalizing with a block of heterochromatin (Supplementary Fig. S10a).

### Macroheterocera

Three members of the Notodontidae and Noctuidae families within the superfamily Noctuoidea were examined. In male pachytene complements of the puss moth, *Cerura* *vinula* (Notodontidae), we verified the diploid chromosome number of 2n = 42 (Supplementary Fig. S2d) previously reported^81^ and observed two autosomal bivalents carrying a terminal rDNA cluster (Supplementary Fig. S9a). One interstitial cluster of histone genes was detected by the histone H3 probe (Supplementary Fig. S9b). In female pachytene nuclei of the buff-tip, *Phalera* *bucephala* (Notodontidae), with a diploid number of chromosomes 2n = 60^81^, the same hybridization pattern for both markers as in *C.* *vinula* was observed (Supplementary Fig. S9c,d). In the fall armyworm, *Spodoptera* *frugiperda* (Noctuidae), with a diploid chromosome number of 2n = 62^81^, only one bivalent bearing an interstitial cluster of rDNA genes was identified by FISH (Supplementary Fig. S9e). Similar to the other two species, one interstitial histone cluster was detected in one of the bivalents in male pachytene nuclei (Supplementary Fig. S9f).

Two representatives of the superfamily Geometroidea were included in our study, the peppered moth *Biston* *betularia* and the magpie moth *Abraxas* *grossulariata* (both Geometridae). In *A.* *grossulariata*, the diploid number of chromosomes 2n = 56 was reported in an earlier study^70^, which also detected a single terminal rDNA cluster on W and Z sex chromosomes. On chromosomal preparations of *A.* *grossulariata,* we identified one interstitial cluster of histone genes (Supplementary Fig. S10b). Moreover, numerous strong DAPI-positive heterochromatin blocks were detected (Supplementary Fig. S10b), which is also in agreement with previous observations^70^. The diploid chromosome number of 2n = 62 was previously reported for *B.* *betularia*^84^ and the same material was used in this study. In male pachytene nuclei, we identified three bivalents bearing a single small terminal rDNA cluster each (Supplementary Fig. S10c). Using the histone H3 probe, a single interstitial histone cluster was detected on one of the autosomal bivalents in female pachytene nuclei (Supplementary Fig. S10d).

Species from three different families were explored within the superfamily Bombycoidea, the drinker moth *Euthrix* *potatoria* (Lasiocampidae), the silkworm *Bombyx* *mori* (Bombycidae), and the cecropia silkmoth *Hyalophora* *cecropia* (Saturnidae). A diploid chromosome number of 2n = 62 was previously described in *E.* *potatoria*^81^ and was confirmed by our results (Supplementary Fig. S2e). Hybridization of the 18S rDNA probe revealed an interesting distribution of rDNA genes, namely two interstitially located clusters within one pachytene bivalent (Supplementary Fig. S11a). The histone H3 probe uncovered one interstitial cluster of histone genes (Fig. S11b). The diploid karyotype of *B.* *mori* consists of 2n = 56 chromosomes^85^. Distribution of rDNA was previously reported as a single interstitial rDNA cluster^29^. We confirmed this in male pachytene preparations (Supplementary Fig. S11c). Moreover, the FISH experiments with the histone H3 probe also revealed an interstitial position of a single histone gene cluster in one of the chromosomal pairs (Supplementary Fig. S11d).

The karyotype of *H.* *cecropia* consists of 2n = 62 chromosomes, as previously reported^86^, which is corroborated also by our observation (Supplementary Fig. S2f). Three terminal clusters of rDNA genes in three different bivalents were mapped by the 18S rDNA probe (Supplementary Fig. S11e). Histone H3 mapping revealed two histone gene clusters at both ends of one bivalent and another cluster at one end of another bivalent colocalizing with strong blocks of heterochromatin. Bivalents bearing the histone clusters were almost exclusively associated in pachytene complements forming a specific configuration (Supplementary Fig. S11f).

### Mapping of 5S rDNA and U1 and U2 snRNA genes

The 5S rDNA gene and U1 and U2 snRNA genes have never been used as cytogenetic markers in the order Lepidoptera. Therefore we decided to test their suitability for comparative analysis within this order. We chose nine species from different families across the whole order Lepidoptera with a focus on basal groups, namely *H.* *humuli* (Hepialidae), *T.* *ekebladella* (Tischeriidae), *T.* *bisselliella* (Tineidae), *T.* *tubulosa* (Psychidae) *C.* *ohridella* (Gracillariidae), *Y.* *evonymella* (Yponomeutidae), *C.* *pomonella* (Tortricidae), *E.* *kuehniella* (Pyralidae), *B.* *mori* (Bombycidae), and one outgroup species, *G.* *pellucidus* (Limnephilidae), from the sister order Trichoptera. We amplified and labelled species-specific probes for the 5S rDNA, U1 and U2 snRNA genes, and used them in FISH experiments in the respective species (Supplementary Table S1).

Although we used an optimized TSA-FISH protocol to maximize sensitivity of the FISH experiments, we successfully mapped 5S rDNA only in four species, namely *G.* *pellucidus*, *H.* *humuli*, *T.* *ekebladella*, and *T.* *tubulosa*. In all four species, we detected one subterminal cluster of 5S rDNA genes on one chromosomal pair (Supplementary Fig. S12). In the other species, no clear hybridization signals were identified. The U1 and U2 snRNA genes did not show any hybridization signals in any of the ten species studied (summary of results in Fig. 1). The negative results of the FISH experiments suggest that the genomic arrangement of these three genes is not suitable for FISH mapping in Lepidoptera. For example, these genes may occur in low numbers in tandem arrays or may be scattered throughout the genome, rather than clustered. To test these hypotheses, we carried out quantitative PCR (qPCR) and Southern hybridization.

### qPCR experiments

**Figure 2:**
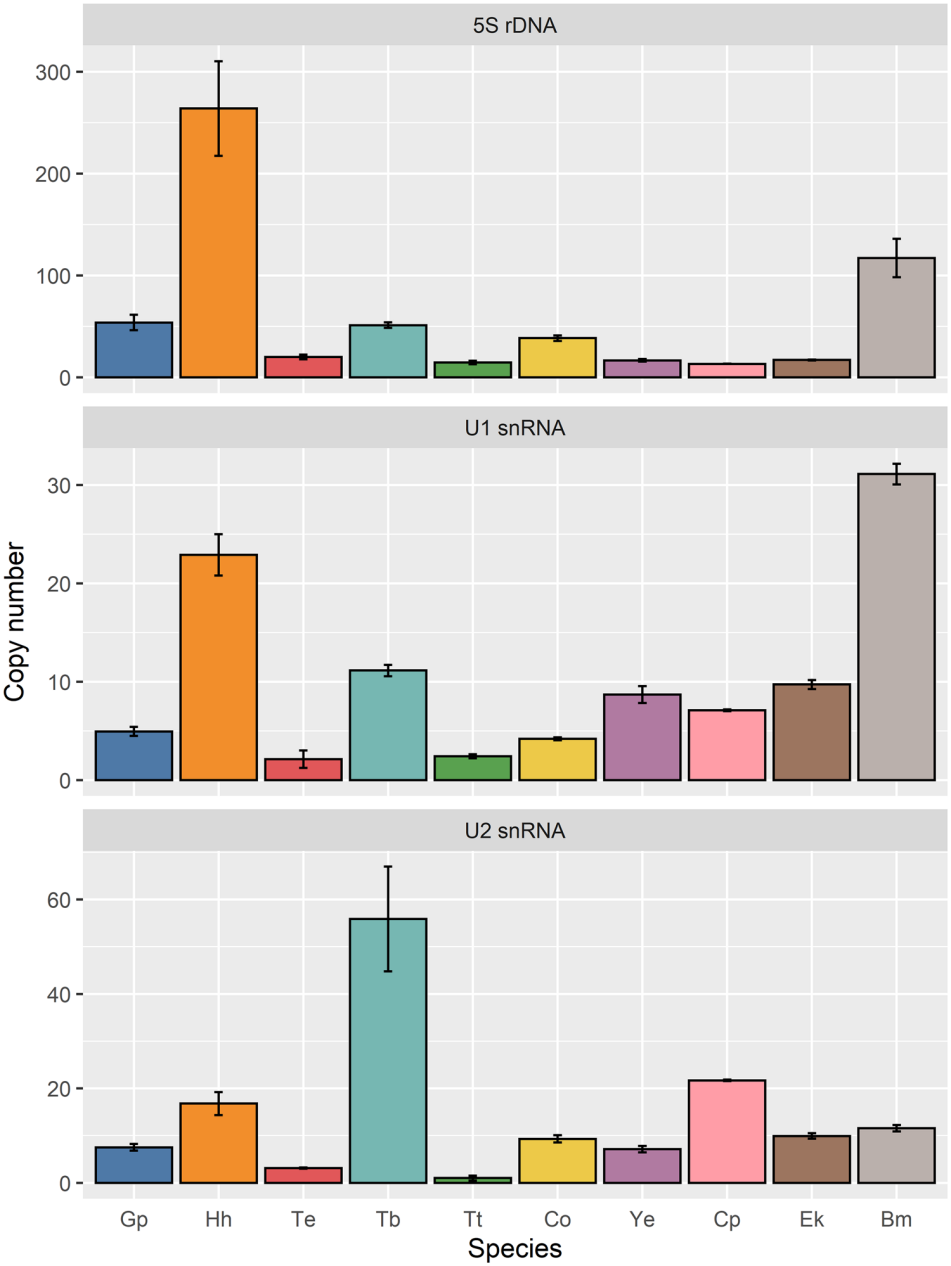
Estimated copy numbers of 5S rDNA, U1 snRNA, and U2 snRNA genes per haploid genome in selected species. Gp, *G.* *pellucidus* (Trichoptera, outgroup); Hh, *H.* *humuli*; Te*,* *T.* *ekebladella*; Tb, *T.* *bisselliella*; Tt, *T.* *tubulosa*; Co, *C.* *ohridella*; Ye, *Y.* *evonymella*; Cp, *C.* *pomonella*; Ek, *E.* *kuehniella*; Bm, *B.* *mori*. For a summary of the qPCR results, see Supplementary Table S3.

Quantitative PCR was carried out to estimate relative copy number of 5S rDNA, U1 snRNA, and U2 snRNA genes in ten representatives probed for these genes by TSA-FISH (see above). Estimated copy numbers are summarized in Fig. 2 and Supplementary Table S3. In the case of 5S rDNA, estimated copy numbers ranging from 13 to 264 copies (mean 60.602 SD ± 14.506, median 29.198). The 5S rDNA copy number was higher (> 50 copies) in three of the four species in which the 5S rDNA locus was detected by TSA-FISH, namely *G.* *pellucidus,* *H.* *humuli,* and *T.* *tubulosa*. Using TSA-FISH we also localized 5S rDNA in *T.* *ekebladella*, although its copy number was much lower (~ 20 copies) and comparable with other species in which 5S rDNA could not be localized. However, we were unable to localize 5S rDNA by TSA-FISH in *C.* *ohridella* and *B.* *mori* with 5S rDNA copy numbers of 38 and > 100, respectively. For U1 snRNA and U2 snRNA, the results obtained showed low copy numbers in all species ranging from 2 to 31 copies (mean 10.436 SD ± 0.595, median 7.885) and from 1 to 56 copies (mean 14.387 SD ± 3.331, median 9.611) per haploid genome, respectively.

### Southern hybridization

To test whether 5S rDNA and U1 snRNA genes are organized in tandem arrays, we performed Southern hybridization in ten selected species (see above). The U2 snRNA was excluded from this analysis due to difficulties in preparing digoxigenin-labelled probes.

Southern hybridization of the 5S rDNA probe was successful in all species tested (Supplementary Fig. S13). Results revealed multiple DNA fragments mostly > 2000 bp bearing the target sequence in all species examined. The intensity of hybridization signals was mostly uniform, although stronger bands correlating with multiple gene copies were identified e.g. in *G.* *pellucidus*, *H.* *humuli*, and *T.* *bisselliella*. Strong bands of smaller size, which presumably correspond to identical repeat units derived from tandem arrays, were observed only in *G.* *pellucidus*. Given the low copy numbers indicated by qPCR, the Southern hybridization results suggest the 5S rRNA gene copies are either scattered throughout the genome or loosely associated, i.e. individual copies are separated by varying spacers longer than 2000 bp.

Hybridization patterns using the U1 snRNA probe were similar to the 5S rDNA patterns in all species tested (Supplementary Fig. S14). Multiple bands of mostly weak intensity were observed, which implies that the 10 copies determined on average by qPCR are mostly either separated by long spacers or scattered across the genome in all species studied. Stronger bands corresponding to DNA fragments bearing multiple U1 snRNA copies were detected e.g. in *C.* *ohridella* and *C.* *pomonella* (Supplementary Fig. S14d,f). In the latter, however, the stronger bands can comprise multiple bands due to insufficient separation of long fragments (Supplementary Fig. S14f). In *T.* *ekebladella* and *T.* *tubulosa*, we were not able to successfully perform Southern hybridization, probably due to the low quality of the input gDNA and/or insufficiently labelled probes.

## Discussion

In this study, we tested whether commonly used cytogenetic markers, namely 18S rDNA, histone H3, 5S rDNA, and U1 and U2 snRNA genes, are applicable and informative for studies of karyotype evolution in Lepidoptera. We employed fluorescence in situ hybridization techniques, rDNA-FISH and TSA-FISH, which enhance hybridization signals by antibody amplification and enzymatically catalysed reporter deposition, respectively. We complemented our FISH results by estimating the copy number of the markers by qPCR and characterizing their genomic organization using Southern hybridization.

Nguyen et al.^29^ reviewed available data on the distribution of major rDNA in Lepidoptera and mapped 18S rDNA in 18 ditrysian species from 4 superfamilies (Pyraloidea, Bombycoidea, Papilionoidea, Noctuoidea). The results suggested that in karyotypes with one locus, rDNA was usually localized interstitially, whereas in karyotypes with two or more clusters, rDNA loci were detected at chromosome ends. It was hypothesized that rDNA can spread to terminal chromosome regions by ectopic recombination between subtelomeric repetitive sequences. However, missing data from non-ditrysian and early diverging ditrysian families did not allow inferring an ancestral rDNA distribution. To fill these gaps, we carried out FISH with the 18S rDNA probe in 27 moth and butterfly species with a special focus on early diverging taxa. We also investigated one trichopteran species as an outgroup.

The results of this study (summarized in the Fig. 1) suggest that one terminal cluster of rDNA genes is an ancestral state, as it is present in the outgroup and across all lepidopteran families. In species with multiple chromosomes bearing rDNA, these clusters are usually located terminally and there is a trend towards an increase in rDNA loci in Lepidoptera. Interestingly, the rDNA loci also multiplied in the early diverging ditrysian lineage Psychidae (Supplementary Fig. S3). The highest numbers of rDNA clusters, 11 and 7, were detected in two nymphalid species, *I.* *io* and *A.* *urticae*, respectively (Supplementary Fig. S7). Other karyotype features, such as chromosomal number n = 31 and presence of a single interstitial histone cluster (Supplementary Fig. S7), which are both considered ancestral traits (Refs.^72,87^; this study, see below), do not point to any large-scale chromosomal rearrangements in these nymphalids. Thus, the multiplication of rDNA clusters in nymphalids concurs with the ectopic recombination-driven spread of rDNA into new loci. Remarkably, multiple interstitial rDNA clusters present on a single bivalent were also documented in some species. Three interstitial rDNA clusters within a single bivalent were detected in *T.* *tubulosa* (Supplementary Fig. S3a) and two interstitial clusters within one bivalent were observed in *E.* *potatoria* (Bombycoidea) (Supplementary Fig. S11a). In both cases, the multiple clusters probably originated from intrachromosomal rearrangements such as inversions of a region containing part of the rDNA cluster (cf. Ref.^88,89^). In the case of *H.* *humuli* (Hepialoidea) (Supplementary Fig. S1c) and *C.* *ohridella* (Gracillarioidea) (Supplementary Fig. S4a), rDNA covers almost half of the chromosome. In addition, in *H.* *humuli* the rDNA cluster colocalizes with a strong heterochromatin block indicating the presence of repetitive sequences potentially associated with rDNA. More detailed research is needed to determine the mechanism of rDNA spread in these two species. Our data show that the multiplication of the major rDNA cluster occurs in multiple lepidopteran families and via different mechanisms, without any clear evolutionary pattern. This erratic behaviour makes the major rDNA an uninformative marker for the study of karyotype evolution in Lepidoptera.

Histone H3 genes have previously been mapped in several lepidopteran species^64,65,90^. One interstitial cluster of histone H3 genes was identified consistently in five species of the family Tortricidae^64^. Histone H3 genes were also localized in four *Leptidea* spp. (Pieridae)^65,90^. The position of the histone gene cluster was stable in *L.* *amurensis*, but in the other three *Leptidea* species, the number and position varied even among the offspring of one female. The karyotype evolution of *Leptidea* butterflies is known to be dynamic, characterized by unstable chromosome numbers^65^, and the distribution of histone gene clusters thus reflects this instability^65,91^. To analyse common trends in histone cluster repatterning across Lepidoptera, we mapped histone H3 genes in 29 moth and butterfly species and one caddisfly outgroup.

In the vast majority of species, TSA-FISH with the histone H3 probe revealed a single bivalent bearing the histone gene cluster (summarized in Fig. 1). This pattern was conserved in several superfamilies, such as Hepialoidea, Tineoidea, Geometroidea, Noctuoidea, and Bombycoidea. In the superfamily Papilionoidea, one interstitial histone gene cluster was observed in nymphalids (Supplementary Fig. S7), whereas in representatives of the family Pieridae, the cluster was identified at the terminal region of a bivalent (Supplementary Fig. S6). This difference in position is most likely the result of an inversion as no additional clusters were identified. This inversion can be one of many chromosomal rearrangements which seem to be typical for the genus *Pieris*^85,92^. A single terminal histone gene cluster was also characteristic of the superfamilies Tischeroidea, Yponomeutoidea, and Cossoidea, although more species need to be tested in these taxa. Multiple clusters were observed only in three species. The caddisfly *G.* *pellucidus* had three terminal clusters on different bivalents (Supplementary Fig. S1b), whereas *T.* *absoluta* (Gelechioidea) had two clusters on both ends of a single bivalent (Supplementary Fig. S8f). In *H.* *cecropia* (Bombycoidea), three terminal clusters were present on two bivalents (Supplementary Fig. S11f). Taken together, the ancestral state of histone genes is probably a single interstitially located cluster. In some taxa, the cluster moved to the chromosome end, allowing its further spread to terminal regions of the same or other chromosomes, probably due to ectopic recombination (cf. Ref.^29^). The localization of the histone gene cluster seems to be very conserved in Lepidoptera, with the exception of *Leptidea* spp.^65^, and its changes indicate chromosomal rearrangements such as inversions, translocations, or chromosomal fusions and fissions (cf. Ref.^65^). Therefore, the histone H3 gene cluster is a good marker to study karyotype evolution in Lepidoptera.

Genes for 5S rRNA and U1 and U2 snRNAs have not yet been localized in lepidopteran genomes. Nine species sampled across Lepidoptera, namely *H.* *humuli* (Hepialidae), *T.* *ekebladella* (Tischeriidae), *T.* *bisselliella* (Tineidae), *T.* *tubulosa* (Psychidae) *C.* *ohridella* (Gracillariidae), *Y.* *evonymella* (Yponomeutidae), *C.* *pomonella* (Totricidae), *E.* *kuehniella* (Pyralidae), and *B.* *mori* (Bombycidae) were analysed along with a trichopteran outgroup, *G.* *pellucidus* (Limnephilidae). The genes and corresponding probes were very short (≤ 140 bp, Supplementary Table S4). Therefore, we used TSA-FISH, which allows the detection of single-copy genes ≥ 1300 bp^27^. Despite the optimization of the protocol, we were unable to localize the U1 and U2 snRNA genes in any of the species studied. The 5S rDNA clusters were detected only in the caddisfly *G.* *pellucidus* and representatives of early diverging lepidopteran lineages, namely *H.* *humuli*, *T.* *ekebladella*, and *T.* *tubulosa*. In all these species, TSA-FISH revealed a single interstitial 5S rDNA cluster (Fig. 1, Supplementary Fig. S12). It is tempting to speculate that the observed phylogenetic pattern could reflect a genome reorganization in Ditrysia, i.e. a lineage comprising 98% of extant moths and butterflies^17^, in which *Hox* gene amplification occurred^93^ and over 1000 novel gene families emerged^94^. However, more data on the distribution of 5S rDNA in early diverging lineages is needed to confirm whether this pattern is consistent.

To find out why 5S rDNA, U1 and U2 snRNAs were not detected by FISH, we determined the copy number of the genes and tested whether the gene copies are arranged in tandem. Quantitative PCR revealed that copy numbers of 5S rRNA genes vary greatly between species (Fig. 2). An upper limit of the 5S rRNA gene copy number was observed in *H.* *humuli*, which may correlate with its likely large genome size. Although the genome size of *H.* *humuli* is unknown, the C-value of other hepialids, *Thitarodes* *(Hepialus)* sp. and *Triodia* *sylvina*, is 2.92 Gb^95^ and 1.8 Gb^96^, respectively. However, copy number alone cannot explain the detectability of 5S rDNA in Lepidoptera. In *T.* *ekebladella*, we found approximately 20 copies of 5S rDNA genes, which we were able to detect by TSA-FISH, while we were not able to detect more than 100 copies of 5S rDNA genes in *B.* *mori.* In all species examined, the total length of the 5S rDNA cluster should be above the detection threshold of 1300 bp^27^, if all the copies are arranged in tandem. However, results of Southern hybridization revealed multiple bands with fragment length > 2000 bp in all species (Supplementary Fig. S13), which suggests that the gene copies are scattered throughout the genome. Indeed, Vierna et al.^43^ reported the presence of ten 5S rDNA clusters in the *B.* *mori* genome based on the analysis of genomic data. Alternatively, the copies can be only loosely clustered, i.e. separated by long spacers varying both in size and sequence. Clusters of 5S rDNA genes have been successfully mapped in many taxa^38,42,97,98^. However, the presence of multiple loci, which remain undetected even by TSA-FISH, questions homology of detected clusters and the usefulness of this marker in studies on karyotype evolution.

Copy number estimates for U1 and U2 snRNA genes by qPCR revealed much lower numbers than for 5S rDNA genes. These differences in copy number between 5S and U1 and U2 snRNAs seem to be consistent in Metazoa^87,99–101^. Based on our Southern hybridization results, the organization of U1 snRNA copies was quite similar to 5S rDNA, as multiple long fragments bearing the studied genes were observed (Supplementary Fig. S14). This means that successful detection of U1 and U2 snRNA clusters by FISH in some taxa^35,46,48,102^ is the exception rather than the rule, and these genes are not universally applicable cytogenetic markers.

Taken together, 5S rDNA, U1 and U2 snRNA genes are not suitable markers for comparative cytogenetic studies in Lepidoptera. With a few exceptions, no clear cluster organization was detected by in situ hybridization. Their scattered organization and/or the presence of long spacer sequences between the genes does not allow for the observation of specific hybridization patterns and thereby the reconstruction of karyotype evolution. On the contrary, hybridization of 18S rDNA and histone H3 genes revealed a clustered organization of these genes in all species studied. Mapping of 18S rDNA showed rather dynamic evolution of the major rDNA, which does not always reflect chromosomal changes. However, various patterns, numbers, and locations of rDNA clusters could provide information on the evolution of repetitive sequences in lepidopteran genomes. Even though the mapping of histone H3 genes requires a species-specific probe preparation, hybridization patterns seem to genuinely reflect chromosomal rearrangements that occurred during the evolution of lepidopteran species. Our study shows that the evaluation of cytogenetic markers can significantly contribute to research focused on comparative cytogenetics and evolutionary genetics not only in Lepidoptera, but in all eukaryotic species.

## Material and methods

### Insects

Examined lepidopteran species and one representative of caddisflies (Trichoptera), which was used as an outgroup, were either collected in the field or obtained from laboratory stocks. Some species were dissected immediately after collection. In the other species, captured females were left to lay eggs in plastic containers with host plants. Hatched larvae were then reared on their host plants or artificial diet. For a list of studied species, their origin, and details of rearing see Table S5.

### Chromosome preparations

Meiotic and mitotic chromosomes from all the studied species were obtained from female and male gonads of 4th or 5th instar larvae. The only exception was *Gonepteryx* *rhamni*, which was dissected as young imago. Chromosomal preparations were made by spreading technique as described previously^103^. Briefly, dissections were performed in physiological solution^104^. The dissected gonads were hypotonized for 10 min (0.075 M KCl) and fixed in Carnoy’s fixative (ethanol, chloroform, acetic acid; 6:3:1) for 15 min. They were then dissociated using tungsten needles in a drop of 60% acetic acid on a slide and spread using a heating plate set at 45 °C. Chromosome preparations were passed through an ethanol series (70%, 80% and 100% ethanol; 30–60 s each) and stored at − 20 °C or − 80 °C until further use.

### FISH with 18S rDNA probe

A partial sequence of 18S rDNA was generated by PCR from male genomic DNA (gDNA) of the codling moth, *Cydia* *pomonella*, using a pair of specific primers as described previously^25^ (Supplementary Table S4). This fragment was ligated into Promega pGem T-Easy Vector (Promega, Madison, WI, USA), cloned, purified by NucleoSpinPlasmid kit (Macherey-Nagel, Düren, Germany), verified by sequencing (SEQme, Dobříš, Czech Republic), and reamplified by PCR from plasmid. The reamplified 18S rDNA fragment was purified by the Wizard SV Gel and PCR Clean-Up System (Promega) and labelled by nick translation using Nick Translation Kit (Abbott Molecular Inc., Des Plaines, IL, USA) for 105 min at 15 °C. The 25 µL labelling reaction contained 500 ng DNA, 40 μM dATP, 40 μM dCTP, 40 μM dGTP, 14.4 μM dTTP, and 25.6 μM biotin-16-dUTP (Roche Diagnostics GmbH, Mannheim, Germany).

FISH experiments were carried out according to the previous study^25^ with some modifications. Briefly, chromosome preparations were removed from the freezer, dehydrated in ethanol series, and air-dried. Preparations were treated with 100 µg/mL RNase A for 1 h at 37 °C to remove RNA and subsequently blocked in 5 × Denhardt’s solution for 30 min at 37 °C. In the next step, the slides were denatured in 70% formamide in 2 × SSC for 3.5 min at 68 °C. After denaturation for 5 min at 90 °C, a probe mixture containing 25 ng of biotin-labelled 18S rDNA probe, 25 µg of sonicated salmon sperm, 50% deionized formamide, 10% dextran sulphate in 2 × SSC in a total volume of 10 µL was applied to the slide and hybridized overnight at 37 °C. The biotin-labelled probe was detected by Cy3-conjugated streptavidin (diluted 1:1000 with blocking solution) (Jackson ImmunoRes. Labs. Inc, West Grove, PA, USA). Signals were amplified with biotinylated anti-streptavidin (diluted 1:25 with blocking solution) (Vector Labs. Inc, Burlingame, CA, USA), which was again detected by Cy3-conjugated streptavidin (diluted 1:1000 with blocking solution). The preparations were counterstained with 0.5 µg/mL of DAPI (4′,6-diamidino-2-phenylindole) and mounted in antifade containing DABCO (1,4-diazabicyclo[2.2.2]octane).

### FISH with tyramide signal amplification (TSA-FISH)

To obtain specific histone H3, 5S rDNA, and U1 and U2 snRNA probes for each species or family, fragments of the respective genes were amplified by PCR using degenerate primers (Supplementary Table S4) and gDNA of each individual species as a template, as detailed previously^64^. Species-specific amplified gene fragments were cloned and verified by sequencing (SEQme) (Supplementary Table S1). The verified plasmids were purified by NucleoSpin Plasmid kit (Macherey–Nagel) and used as template DNA to prepare a labelled probe by PCR. Each 25 µL labelling reaction contained 1–10 ng template DNA, 1 × Ex *Taq* buffer, 1 mM each dATP, dCTP, and dGTP; 0.36 mM dTTP; 0.64 mM of fluorescein-12-dUTP (PerkinElmer, Waltham, MA, USA), 5 µmol of each primer, and 0.25 U TaKaRa Ex *Taq* DNA polymerase (TaKaRa, Otsu, Japan). Labelled probes were purified using Sephadex (Illustra Sephadex G-50 fine DNA grade). In *Inachis* *io* and *Tuta* *absoluta,* rDNA clusters were also mapped by TSA FISH with 18S rDNA probe labelled by fluorescein instead of FISH with biotin-labelled probe described above. Species-specific probes were generated for most species, except for *Phymatopus* *californicus*, to which we hybridized a probe from *Hepialus* *humuli*, *Psyche* *crassiorella* with a probe from *Taleporia* *tubulosa*, and *Pieris* *brassicae* with a probe from *Pieris* *rapae*.

TSA-FISH was performed according to the published protocol^27^ with some modifications. Briefly, frozen chromosome preparations were dehydrated using an ethanol series. After drying, slides were treated with 10 mM HCl for 10 min at 37 °C to remove cytoplasm and incubated in 1% hydrogen peroxide for 30 min at room temperature to quench endogenous peroxidase activity. Preparations were digested with 100 µg/mL RNase A for 1 h at 37 °C and blocked with 5 × Denhardt’s solution for 30 min at 37 °C. Thereafter, a 50 µL probe mixture containing 10–30 ng of labelled specific probe in 50% deionized formamide and 10% dextran sulfate in 2 × SSC was added to the slide, and the probe and chromosomes were simultaneously denatured for 5 min at 70 °C. Hybridization took place overnight at 37 °C. Hybridization signals were enhanced by Antifluorescein-HRP conjugate (PerkinElmer) diluted 1:1000 and incubated with tyramide solution (TSA Plus Fluorescein system, PerkinElmer) for 10–15 min for 5S rDNA and U1 and U2 snRNA and 5–7 min for histone H3. The preparations were counterstained and mounted in antifade containing DABCO with 0.5 µg/mL of DAPI.

### Microscopy and image processing

Observation of chromosome preparations from FISH experiments was performed with a Zeiss Axioplan 2 microscope (Carl Zeiss, Jena, Germany) equipped with appropriate fluorescence filter sets. An Olympus CCD monochrome camera XM10 equipped with cellSens 1.9 digital imaging software (Olympus Europa Holding, Hamburg, Germany) was used to record and capture black-and-white pictures. Images were captured separately for each fluorescent dye and then pseudocoloured and superimposed with Adobe Photoshop CS4, version 11.0.

### Quantitative analysis of gene doses

Quantitative PCR (qPCR) was used to estimate relative copy numbers of three target genes, namely U1 and U2 snRNA and 5S rDNA, in *Glyphotaelius* *pellucidus*, *Hepialus* *humuli*, *Tischeria* *ekebladella*, *Taleporia* *tubulosa,* *Tineola* *bisselliella,* *Cameraria* *ohridella*, *Yponomeuta* *evonymella,* *Ephestia* *kuehniella*, *Cydia* *pomonella*, and *Bombyx* *mori*^19,76^. By comparing the genes of interest to a single-copy autosomal reference gene (*Acetylcholinesterase* *2*, *Ace2*), their relative copy numbers were estimated based on a target to reference gene dose ratio formula (Ref.^105^; see below). The reference gene and genes of interest were analysed simultaneously in technical triplicates of three independent biological replicas. Due to small body size of some species, namely *T.* *ekebladella*, *T.* *tubulosa,* *T.* *bisselliella*, and *C.* *ohridella*, 5–10 individuals were pooled for gDNA extraction carried out using NucleoSpin Tissue kit (Macherey–Nagel), DNeasy Blood & Tissue Kit (Qiagen, Hilden, Germany), or NucleoSpin DNA Insect kit (Macherey–Nagel) following manufacturer’s instructions. One individual per biological replica was used for the other species.

The qPCR contained 1–10 ng of gDNA, an optimized concentration of primers per species (details in Supplementary Table S6) and Xceed qPCR SG Mix Lo-ROX (Institute of Applied Biotechnologies, Prague, Czech Republic) in a total volume of 10 µL. Amplification efficiencies (*E*) for each gene and species were determined by 0×, 5×, 25×, and 125× dilutions of pooled gDNA of all biological replicas. For all three markers in *C.* *pomonella* and 5S rDNA in *B.* *mori* SYBR Premix Ex *Taq* II Perfect Real Time (1×; TaKaRa) was used and amplification efficiencies were determined by 0×, 10×, 100× and 1000× dilutions (details in Supplementary Table S6). The experiments were carried out using the C1000 Thermal cycler CFX96 Real-Time System (Bio-Rad, Hercules, CA, USA) and data were analysed using software Bio-Rad CFX Manager 3.1. The target to reference gene dose ratio was calculated for each biological sample according the formula *R* = [(1 + *E*_Reference_)^CtReference^]/[(1 + *E*_Target_)^CtTarget^], where *R* is a relative copy number of target gene, *E* is the primer efficiency and Ct = cycle threshold^105^.

### Southern hybridization

Southern hybridization was performed to independently estimate the copy number of U1 snRNA and 5S rDNA and to test whether the genes are tandemly arranged in the genomes of ten selected species. Cloned fragments of studied genes were reamplified by PCR using degenerate primers (Supplementary Table S4) and the products were used as template for labelling with digoxigenin-11-dUTP (Roche Diagnostics GmbH). Labelling and purification of the probes were done as for TSA-FISH probes (see above).

High-molecular-weight gDNA of the studied species was extracted by standard phenol–chloroform^106^ or by cetyltrimethylammonium bromide (CTAB)^107^ extraction. Three pairs of restriction enzymes with no restriction sites within the target sequences were selected (Supplementary Table S7) and digestion of gDNA was carried out overnight at 37 °C. Enzymes were inactivated by addition of loading buffer (50% glycerol, 250 mM EDTA, 5.9 mM bromophenol blue) or Gel Loading Dye, Purple (6×) (New England Biolabs, Ipswich, MA, USA) (for details see Supplementary Table S7). Five micrograms of digested DNA per well was separated using a 1% agarose gel in 1 × TBE buffer by horizontal electrophoresis at 5 V/cm. Southern hybridizations were carried out according to the published protocol^108^ with some modifications. Briefly, after electrophoretic separation, DNA was denatured and transferred onto an Amersham Hybond-N + nylon membrane (GE Healthcare, Buckinghamshire, UK) by capillary flow. Hybridization of labelled probes (100 ng) was done overnight at 42 °C and the stringent washes on the subsequent day were performed at 68 °C. Probes were detected using Anti-Digoxigenin-AP (75 mU/mL; Roche Diagnostics GmbH) incubated with CDP-*Star* ready-to-use (Roche Diagnostics GmbH). Resulting chemiluminescence was recorded with a LAS-3000 Lumi-Imager (Fuji Photo Film Europe GmbH, Düsseldorf, Germany).

## Supplementary Information

Supplementary Information.

## Data Availability

All data generated or analysed during this study are included in this published article [and its Supplementary Information files]. Partial sequences of genes under study were deposited in GenBank under the acc. nos. MW149037–MW149046, MW194851–MW194870, and MW558903–MW558929.
