# Supplementary material for: Large-scale comparative analysis of cytogenetic markers across Lepidoptera
Source: Sci Rep. 2021 Jun 9;11:12214. doi: 10.1038/s41598-021-91665-7 (PMC8190105; doi:10.1038/s41598-021-91665-7)
Supplement: Supplementary file 1 — Supplementary Information. [file 41598_2021_91665_MOESM1_ESM.pdf]

Supplementary Figures and Tables for

Large-scale comparative analysis of cytogenetic markers across Lepidoptera

by

Irena Provazníková, Martina Hejníčková, Sander Visser, Martina Dalíková, Leonela Z. Carabajal Paladino, Magda Zrzavá, Anna Voleníková, František Marec, and Petr Nguyen*

* Correspondence to:
Petr Nguyen
petr.nguyen@prf.jcu.cz

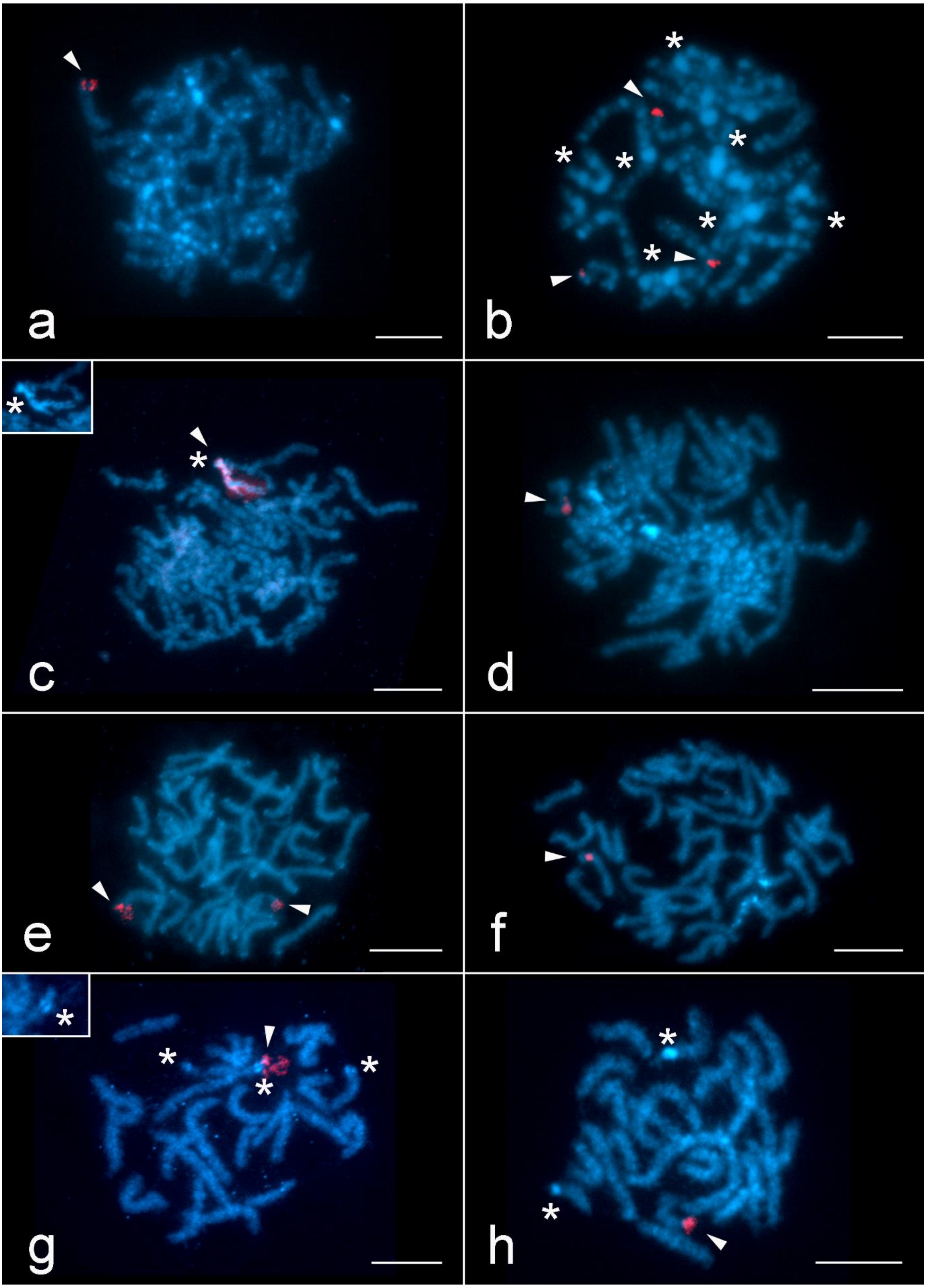

Supplementary Figure 1: Major rDNA and histone H3 clusters detected by rDNA FISH and TSA-FISH, respectively, on pachytene nuclei of representatives of the caddisfly superfamily Limnephiloidea (Trichoptera) and lepidopteran superfamilies Hepialoidea and Tischeroidea. **a**, **c**, **e**, **g** – FISH with 18S rDNA probe (red signals marked by arrowheads); **b**, **d**, **f**, **h** – TSA-FISH with histone H3 probe (red

signals marked by arrowheads). Chromosomes are counterstained with DAPI (blue). **a**, **b** – pachytene nuclei of the caddisfly *Glyphotaelius pellucidus* (sex not determined); **c**, **d** – male pachytene nuclei of *Hepialus humuli*, note that the rDNA signals cover approximately half of one of the chromosomal bivalents and colocalize with heterochromatin (C and inset); **e** – female pachytene nucleus of *Phymatopus californicus,* **f** – male pachytene nucleus of *P. californicus*; **g**, **h** – male pachytene nuclei of *Tischeria ekebladella*, note that the cluster of rDNA signals is adjacent to a pair of heterochromatin blocks (**g** and inset)*.* * DAPI positive heterochromatin. Scale bar = 10 µm.

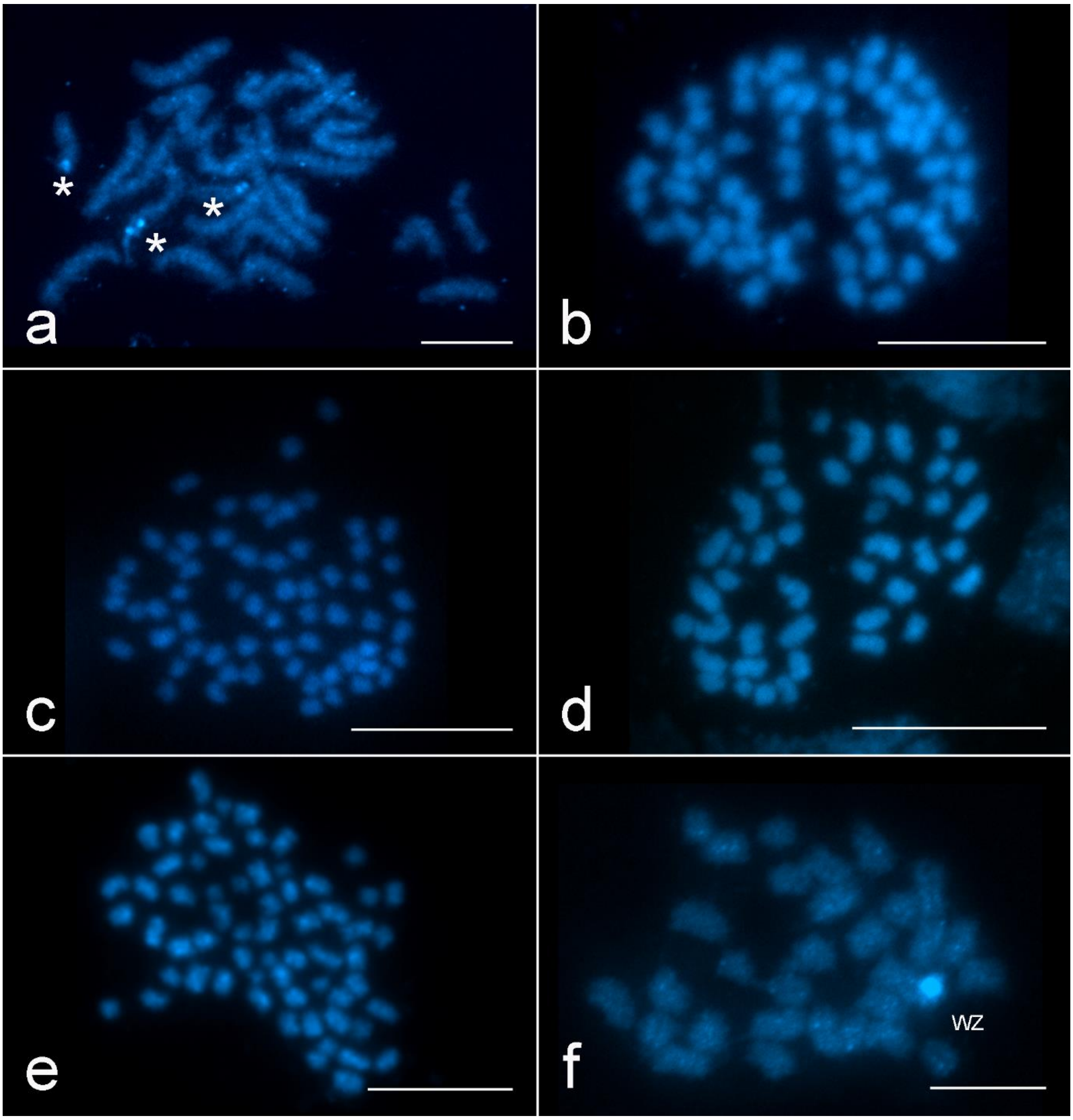

Supplementary Figure 2: Karyotypes of species with previously reported chromosome numbers. Chromosomes are counterstained by DAPI (blue). **a** – male pachytene nucleus of *Tischeria ekebladella* with 23 bivalents and DAPI positive heterochromatin marked by asterisks; **b** – male mitotic nucleus of *Aglais urticae* consisting of 2n=62 chromosomes; **c** - female mitotic nucleus of *Inachis io* comprising 2n=62; **d** - male mitotic nucleus of *Cerura vinula* comprising 2n=42 chromosomes; **e** - male mitotic nucleus of *Euthrix potatoria* comprising 2n=62 chromosomes; **f** - female post-pachytene nucleus of *Hyalophora cecropia* nurse cell with n= 31 bivalents; note DAPI positive W chromosome. Scale bar = 10 µm.

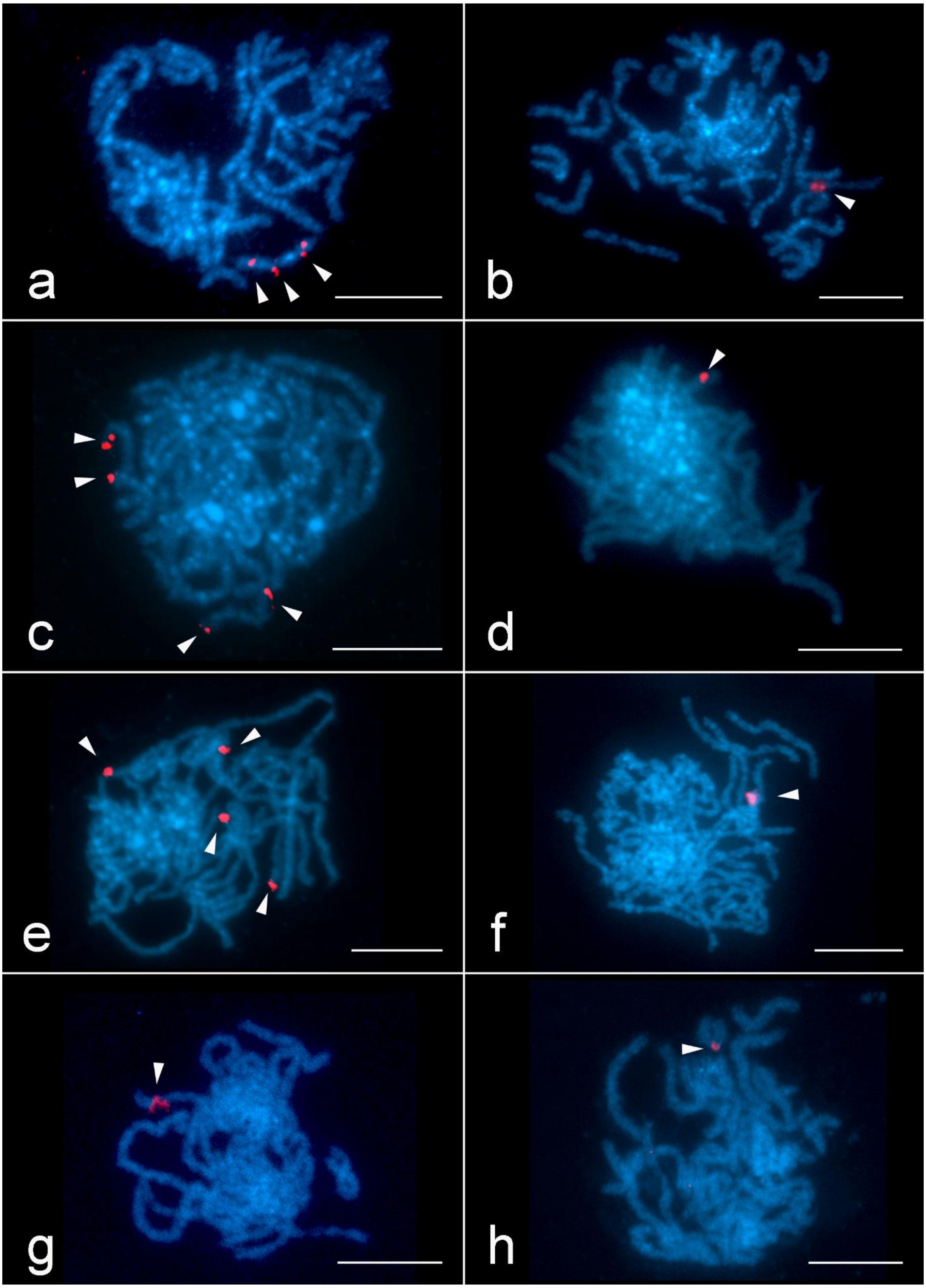

Supplementary Figure 3: Major rDNA and histone H3 clusters detected by rDNA FISH and TSA-FISH, respectively, on pachytene nuclei of four representatives of the superfamily Tineoidea. **a**, **c**, **e**, **g**– FISH with 18S rDNA probe (red signals marked by arrowheads); **b**, **d**, **f**, **h** – TSA FISH with histone H3 probe (red signals marked by arrowheads). Chromosomes are counterstained with DAPI (blue). **a**, **b** –

female pachytene nucleus of *Taleporia tubulosa*; **c**, **d** – male pachytene nucleus of *Proutia betulina*; **e** – male pachytene nucleus of *Psyche crassiorella*, **f** – female pachytene nucleus of *P. crassiorella*; **g**, **h** – male pachytene nucleus of *Tineola bisselliella*. Scale bar = 10 µm.

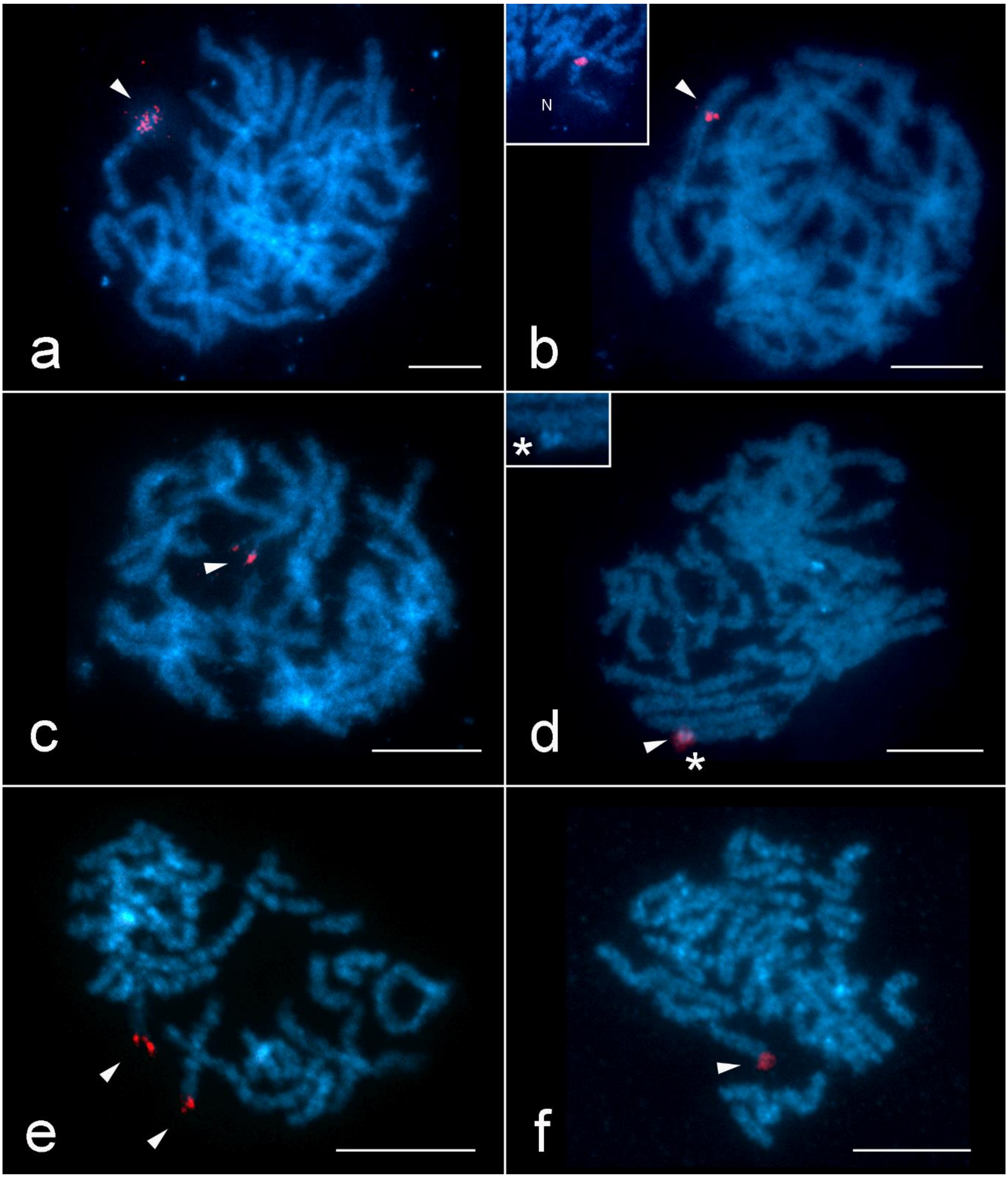

Supplementary Figure 4: Major rDNA and histone H3 loci detected by rDNA FISH and TSA-FISH, respectively, on pachytene nuclei of representatives of the superfamilies Gracillaroidea and Yponomeutoidea. **a**, **c**, **e** – FISH with rDNA probe (red signals marked by arrowheads); **b**, **d**, **f** – TSA-FISH with histone H3 probe (red signals marked by arrowheads). Chromosomes are counterstained with DAPI (blue). **a**, **b** – male nucleus of *Cameraria ohridella*, note the visible nucleolus (N) and histone H3 signal present on the same chromosome pair (**b**, inset); **c**, **d** – male nucleus of *Plutella xylostella,* note that the histone H3 locus is associated with a pair of heterochromatin blocks (**d** and inset).; **e**, **f** – female pachytene nucleus of *Yponomeuta evonymella*. * DAPI positive heterochromatin. Scale bar = 10 µm.

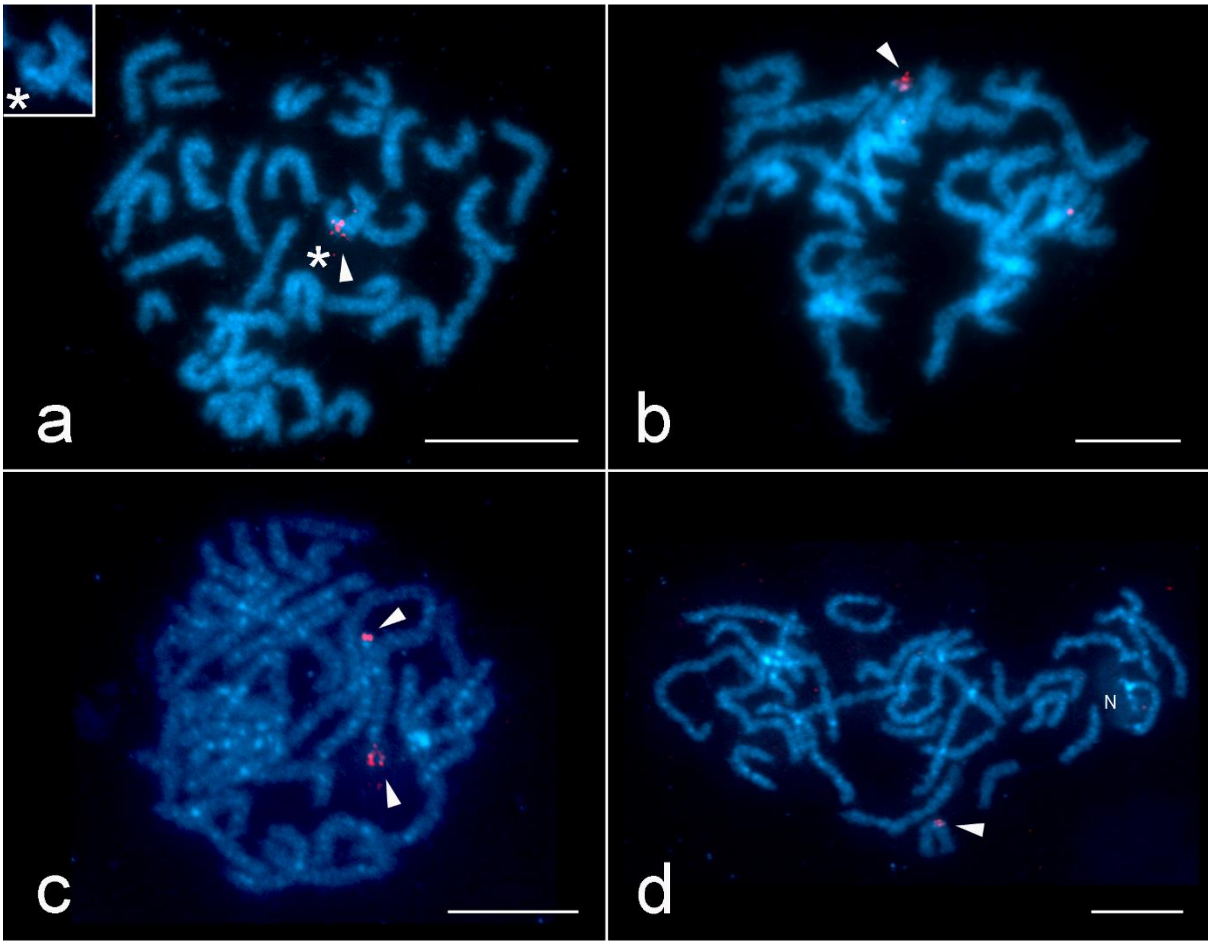

Supplementary Figure 5: Major rDNA and histone H3 clusters detected by rDNA FISH and TSA-FISH, respectively, on pachytene nuclei of representatives of the superfamilies Cossoidea and Tortricoidea. **a**, **c** – FISH with 18S rDNA probe (red signals marked by arrowheads); **b**, **d** – TSA-FISH with histone H3 probe (red signals marked by arrowheads). Chromosomes are counterstained with DAPI (blue). **a** – male pachytene nucleus of *Cossus cossus* with 30 visible chromosomal pairs (2n=60), note that 18S rDNA signal colocalizes with heterochromatin (**a** and inset) ; **b** – male pachytene nucleus of *C. cossus*; **c**, **d** – male pachytene nuclei of *Cydia pomonella*. * DAPI positive heterochromatin. N – nucleolus. Scale bar = 10 µm.

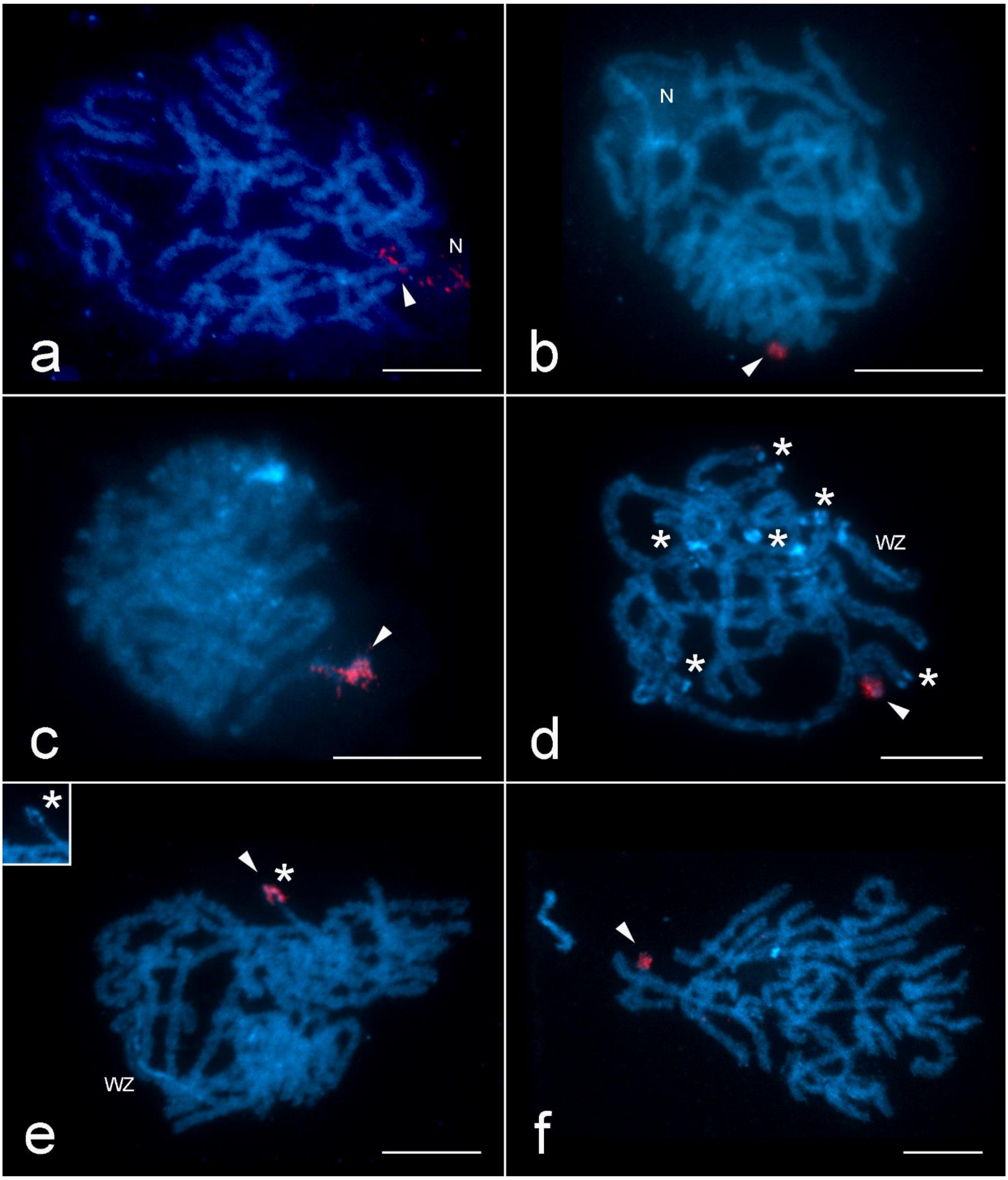

Supplementary Figure 6: Major rDNA and histone H3 clusters detected by rDNA FISH and TSA-FISH, respectively, on pachytene nuclei of three representatives of the family Pieridae (Papilionoidea). **a**, **c**, **e** – FISH with 18S rDNA probe (red signals marked by arrowheads); **b**, **d**, **f** – TSA-FISH with histone H3 probe (red signals marked by arrowheads). Chromosomes are counterstained with DAPI (blue). **a**, **b** – male pachytene nucleus of *Pieris rapae*; **c**, **d** – female pachytene nucleus of *Pieris brassicae*; **e**, **f** – female pachytene nucleus of *Gonepteryx rhamni,* note that 18S rDNA signal colocalizes with heterochromatin (**e** and inset); WZ – sex chromosome bivalent. N – nucleolus. * DAPI positive heterochromatin. Scale bar = 10 µm.

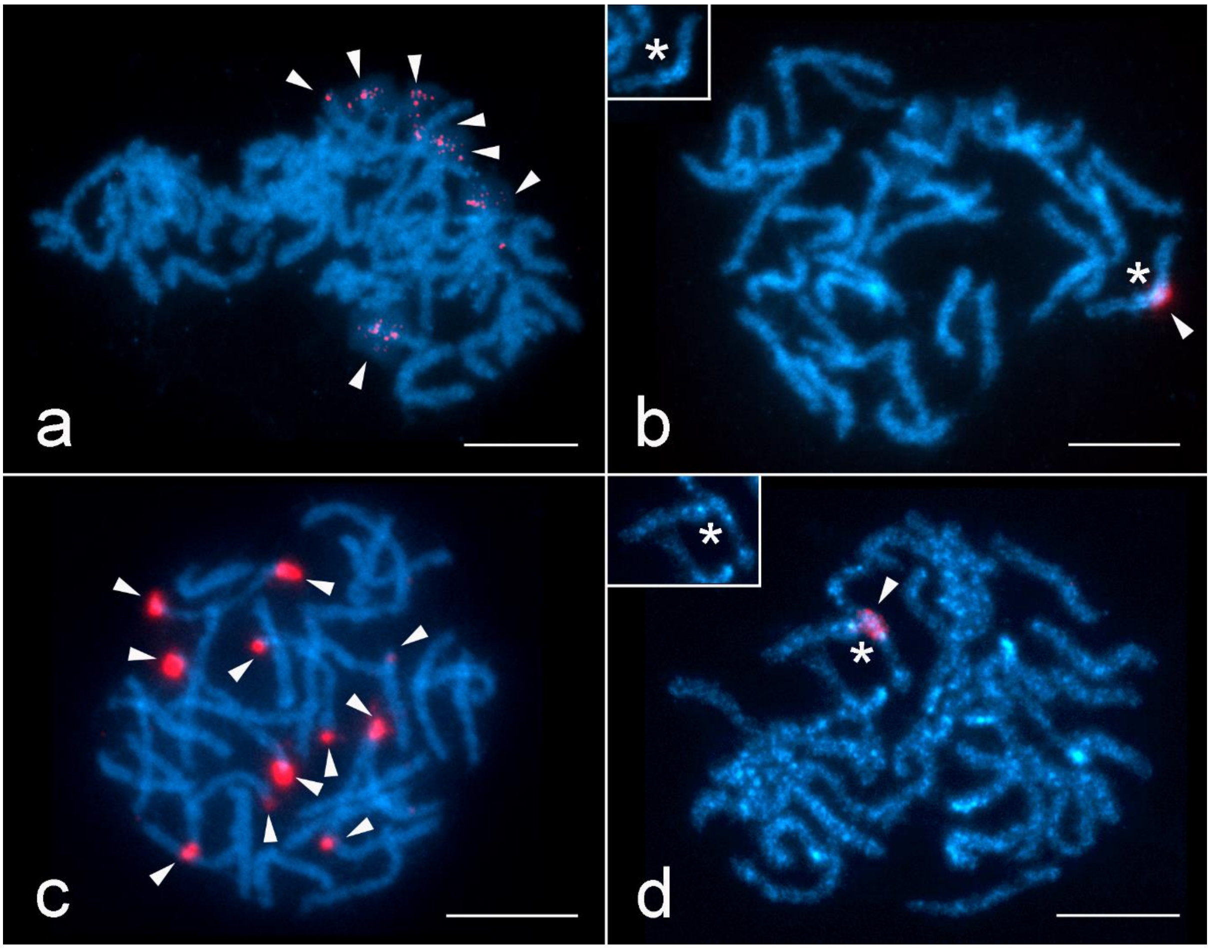

Supplementary Figure 7: Major rDNA and histone H3 clusters detected by rDNA FISH and TSA-FISH, respectively, on pachytene nuclei of two representatives of the family Nymphalidae (Papilionoidea). **a**, **c** – FISH with 18S rDNA probe (red signals marked by arrowheads); **b**, **d** – TSA-FISH with histone H3 probe (red signals marked by arrowheads). Chromosomes are counterstained with DAPI (blue). **a** – male pachytene nucleus of *Aglais urticae*, **b** – female pachytene nucleus of *A. urticae*, note that histone H3 signal colocalizes with heterochromatin (**b** and inset); **c**, **d** – female pachytene nuclei of *Inachis io*, note that histone H3 signal colocalizes with heterochromatin (**d** and inset). * DAPI positive heterochromatin. Scale bar = 10 µm.

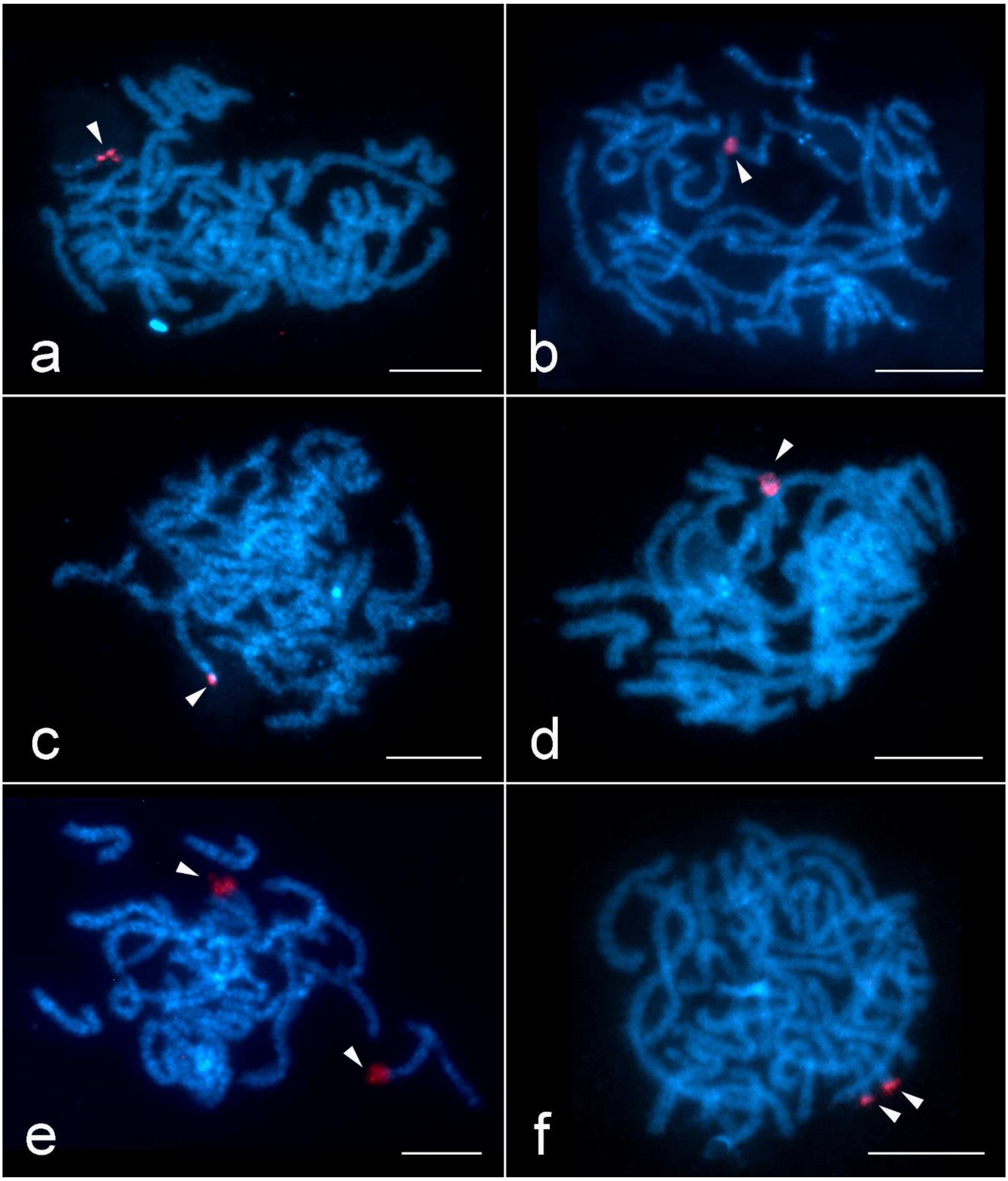

Supplementary Figure 8: Major rDNA and histone H3 clusters detected by rDNA FISH and TSA-FISH, respectively, on pachytene nuclei of three representatives of the superfamily Gelechioidea. **a**, **c**, **e** – FISH with 18S rDNA probe (red signals marked by arrowheads); **b**, **d**, **f** – TSA-FISH with histone H3 probe (red signals marked by arrowheads). Chromosomes are counterstained with DAPI (blue). **a**, **b** – male pachytene nuclei of *Depressaria daucella*; **c**, **d** – male pachytene nuclei of *Limnaecia phragmitella,* note that the 18S rDNA signal colocalizes with heterochromatin (**c**); **e**, **f** – male pachytene nuclei of *Tuta absoluta*. Scale bar = 10 µm.

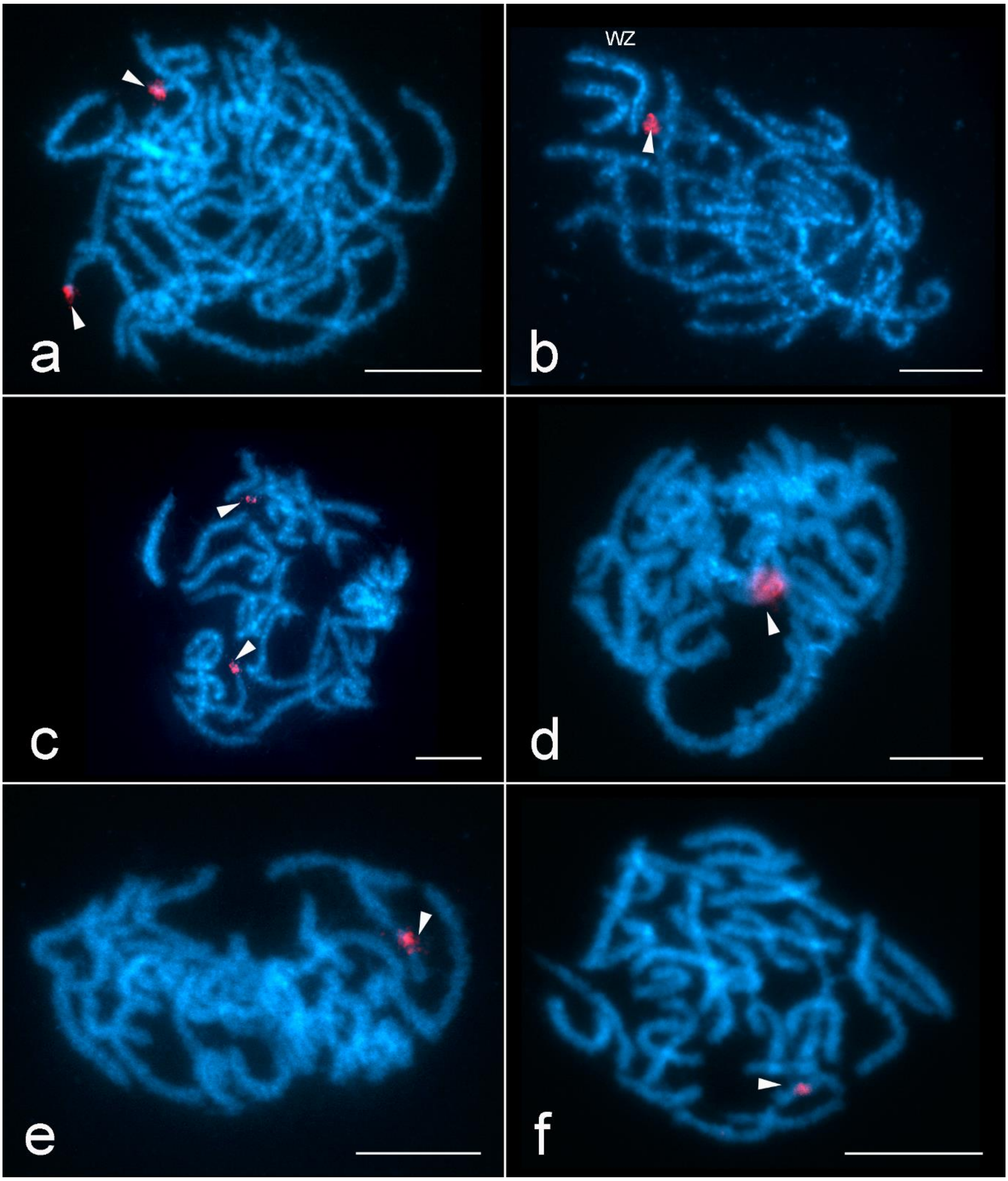

Supplementary Figure 9: Major rDNA and histone H3 clusters detected by rDNA FISH and TSA-FISH, respectively, on pachytene nuclei of three representatives of the superfamily Noctuoidea. **a**, **c**, **e** – FISH with 18S rDNA probe (red signals marked by arrowheads); **b**, **d**, **f** – TSA-FISH with histone H3 probe (red signals marked by arrowheads). Chromosomes are counterstained with DAPI (blue). **a** – male pachytene nucleus of *Cerura vinula*, **b** – female pachytene nucleus of *C. vinula*; **c**, **d** – female pachytene nuclei of *Phalera bucephala*; **e**, **f** – male pachytene nuclei of *Spodoptera frugiperda*. WZ – sex chromosome bivalent. Scale bar = 10 µm.

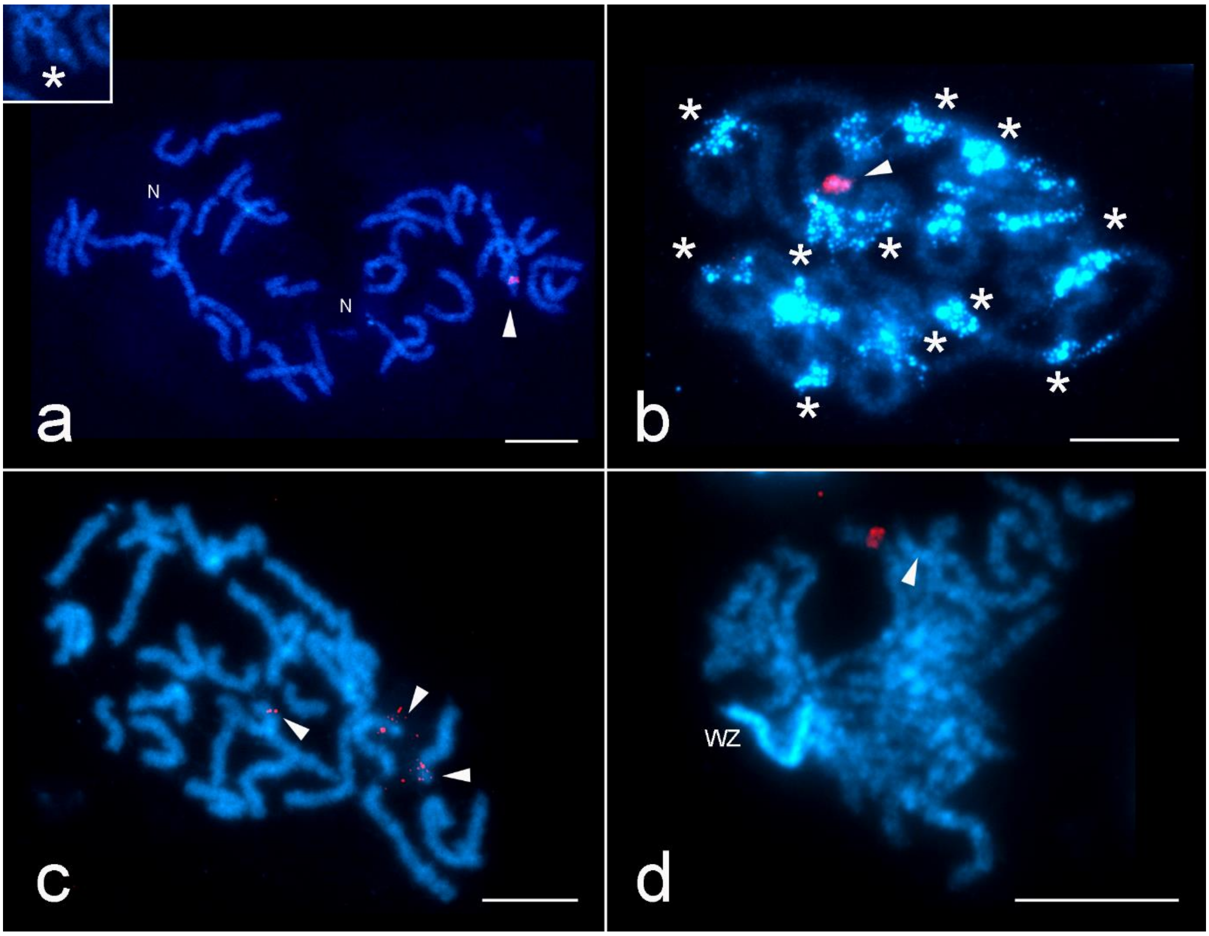

Supplementary Figure 10: Major rDNA and histone H3 clusters detected by rDNA FISH and TSA-FISH, respectively, on pachytene nuclei of representatives from superfamilies Pyraloidea and Geometroidea. **c** – FISH with 18S rDNA probe (red signals marked by arrowheads); **a**, **b**, **d** – TSA-FISH with histone H3 probe (red signals marked by arrowheads). Chromosomes are counterstained with DAPI (blue). **a** – male pachytene nucleus of *Ephestia kuehniella* with two visible nucleoli (N), note that histone H3 signal colocalizes with heterochromatin (**a**, inset); **b** – male pachytene nucleus of *Abraxas grossulariata*; **c** – male pachytene nucleus of *Biston betularia*, **d** – female pachytene nucleus of *B. betularia*. WZ – sex chromosome bivalent. * DAPI positive heterochromatin. Scale bar = 10 µm.

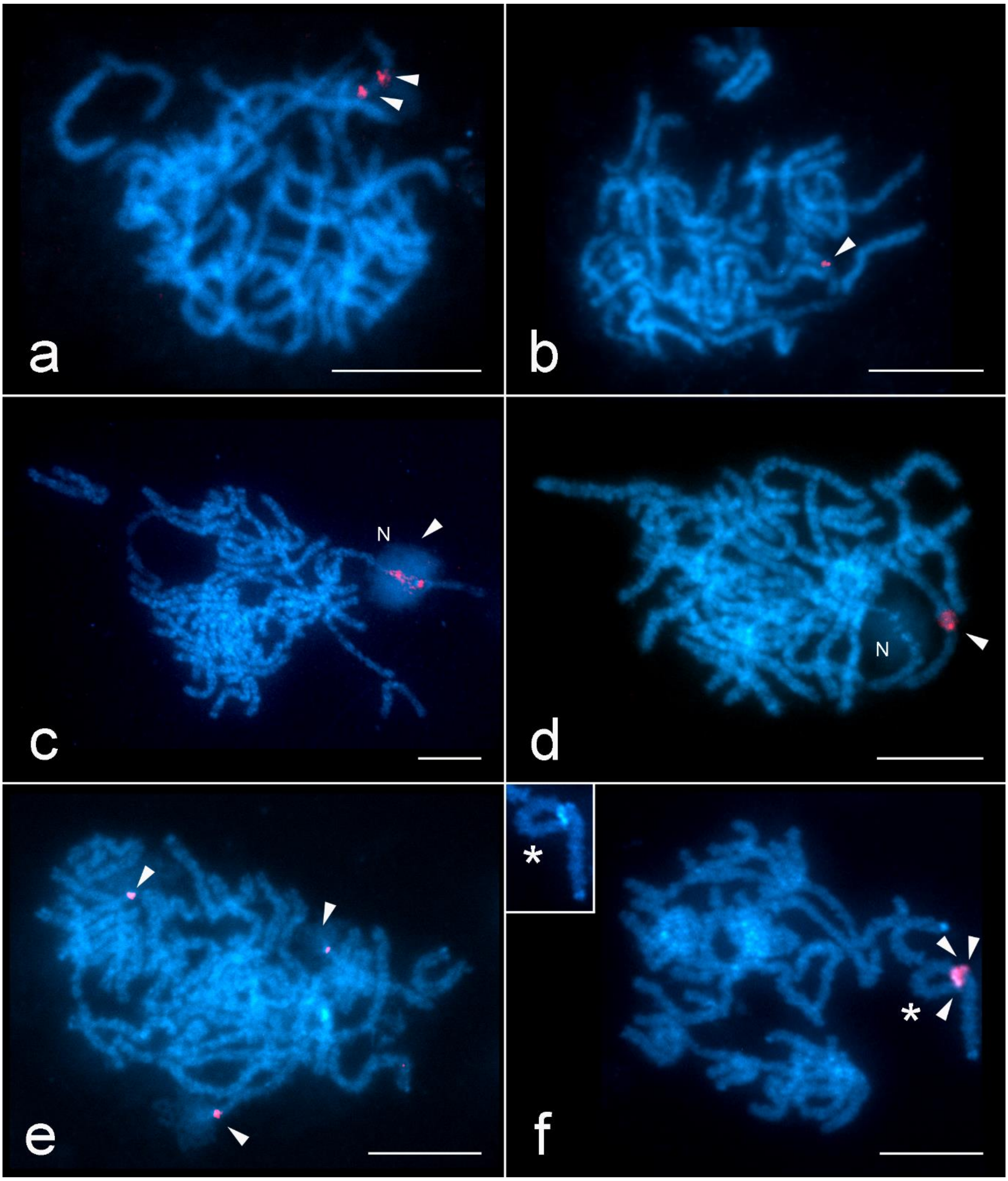

Supplementary Figure 11: Major rDNA and histone H3 clusters detected by rDNA FISH and TSA-FISH, respectively, on pachytene nuclei of three representatives of the superfamily Bombycoidea. **a**, **c**, **e** – FISH with 18S rDNA probe (red signals marked by arrowheads); **b**, **d**, **f** – TSA-FISH with histone H3 probe (red signals marked by arrowheads). Chromosomes are counterstained with DAPI (blue). **a**, **b** – male pachytene nuclei of *Euthrix potatoria*; **c**, **d** – male pachytene nuclei of *Bombyx mori* with visible nucleolus (N); **e** – male pachytene nucleus of *Hyalophora cecropia*, **f** – female pachytene nucleus of *H. cecropia,* note that the histone H3 signals colocalize with heterochromatin (**f**, inset). N – nucleolus. * DAPI positive heterochromatin. Scale bar = 10 µm.

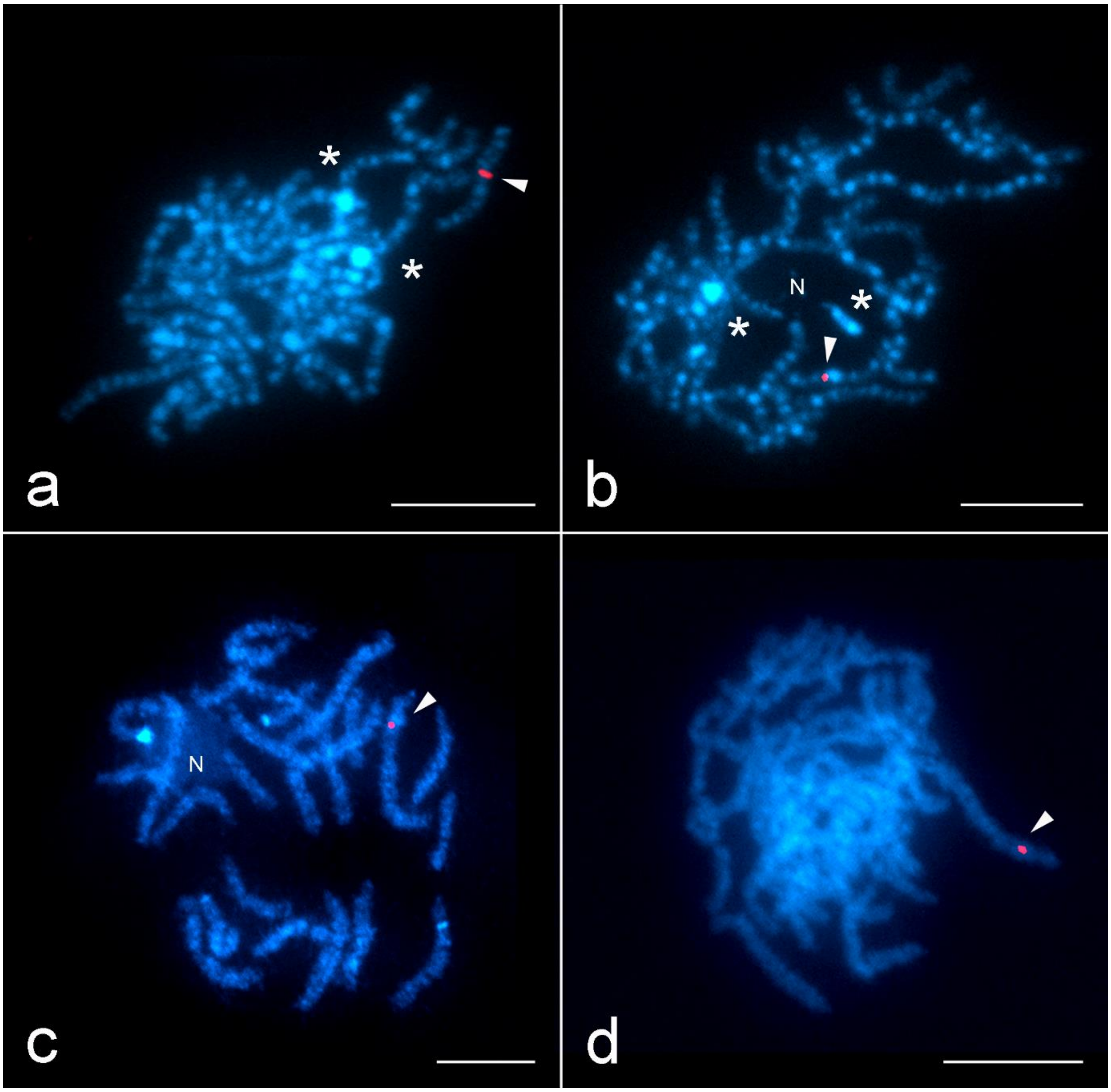

Supplementary Figure 12: 5S rDNA clusters detected by TSA-FISH on pachytene nuclei of representatives of the caddisfly superfamily Limnephiloidea (Trichoptera) and lepidopteran superfamilies Hepialoidea, Tischeroidea, and Tineoidea. TSA-FISH with 5S rDNA probe (red signals and arrowheads). Chromosomes are counterstained with DAPI (blue). **a** – pachytene nucleus of *Glyphotaelius pellucidus* (sex not determined), **b** – male pachytene nucleus of *Hepialus humuli* with visible nucleolus (N), **c** – male pachytene nucleus of *Tischeria ekebladella* with visible nucleolus (N), **d** – male pachytene nucleus of *Taleporia tubulosa*. * DAPI positive heterochromatin. Scale bar = 10 µm.

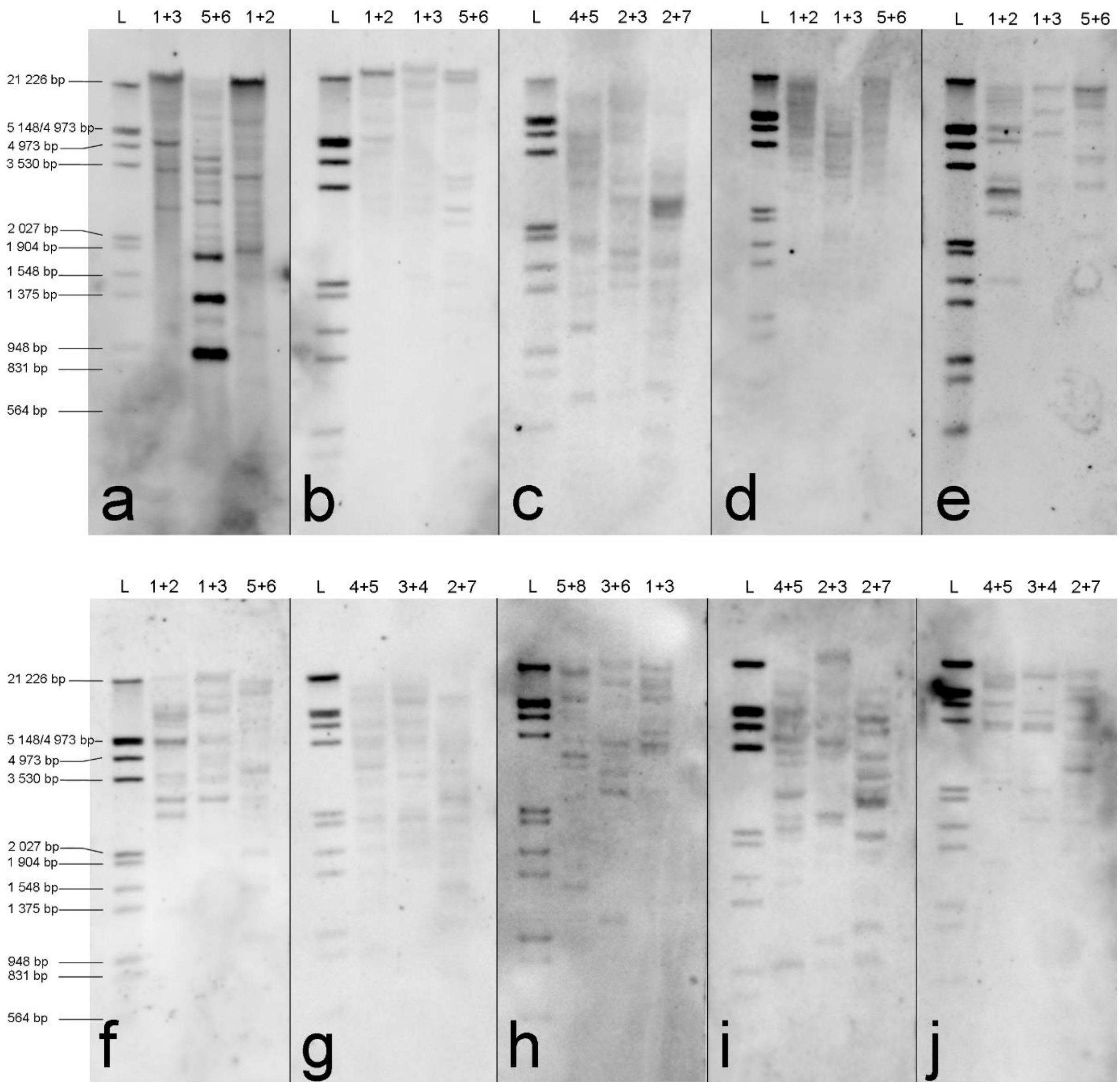

Supplementary Figure 13: Results of Southern hybridization of 5S rDNA probes with genomic DNAs of ten selected species. **a** – *Glyphotaelius pellucidus*, **b** – *Hepialus humuli*, **c** – *Tischeria ekebladella*, **d** – *Taleporia tubulosa*, **e** – *Tineola bisselliella*, **f** – *Cameraria ohridella,* **g** – *Yponomeuta evonymella*, **h** – *Cydia pomonella,* **i** – *Ephestia kuehniella*, **j** – *Bombyx mori.* **L** – DNA molecular weight marker III (Roche Diagnostics GmbH). Restriction enzymes: **1** *Afe*I, **2** *Xba*I, **3** *Bam*HI, **4** *Bsp*HI, **5** *Pst*I, **6** *Sal*I, **7** *Nsp*I, **8** *Not*I. For details on enzymatic digestion reactions, see Table S7.

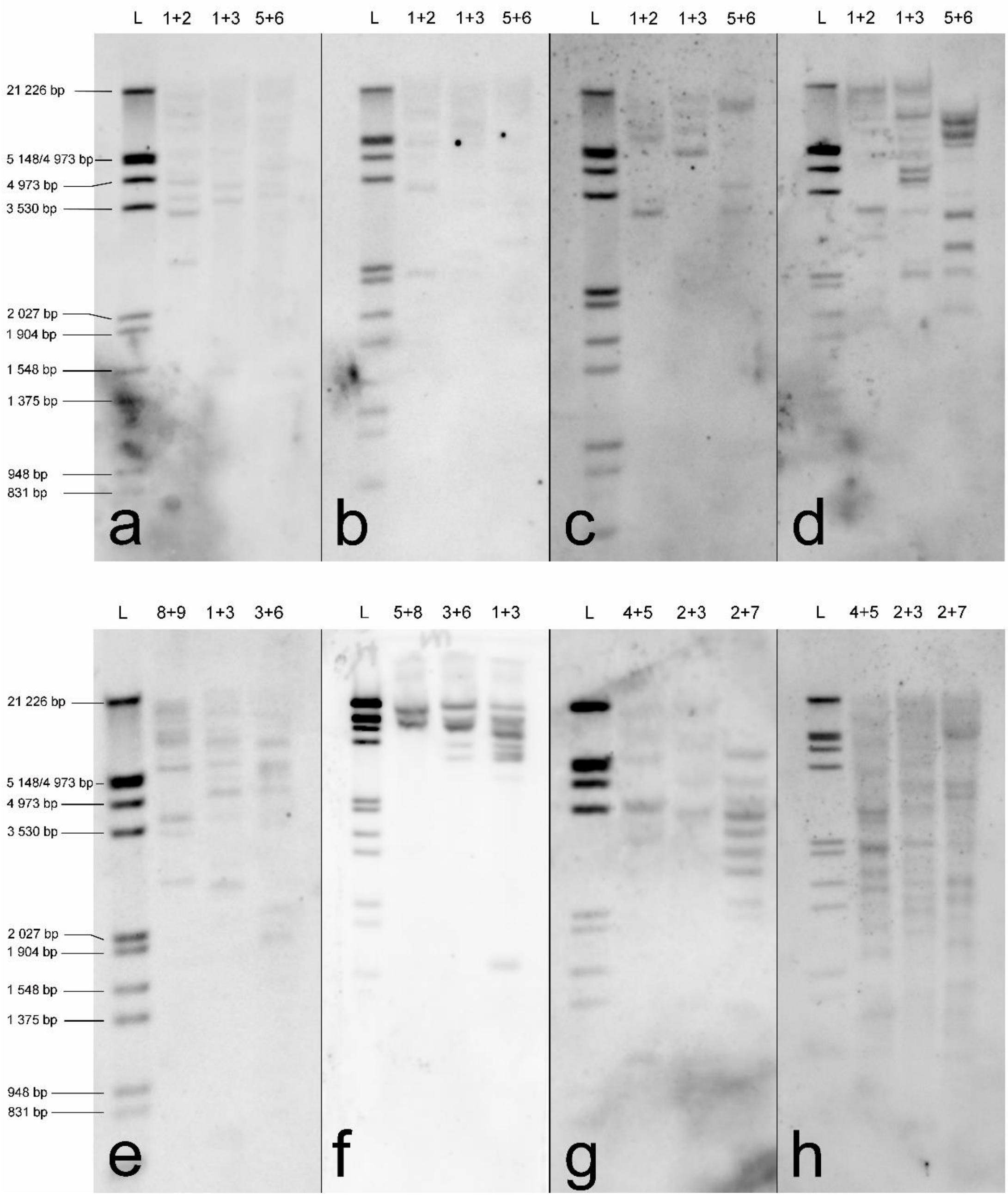

Supplementary Figure 14: Results of Southern hybridisation of U1 snRNA probes with genomic DNA of selected species. Note that multiple bands of varying intensity can be seen in most species. **a** – *Glyphotaelius pellucidus*, **b** – *Hepialus humuli*, **c** – *Tineola bisselliella*, **d** – *Cameraria ohridella,* **e** – *Yponomeuta evonymella*, **f** – *Cydia pomonella,* **g** – *Ephestia kuehniella*, **h** – *Bombyx mori.* **L** – DNA molecular weight marker III (Roche Diagnostics GmbH). Restriction enzymes: **1** *Afe*I, **2** *Xba*I, **3** *Bam*HI, **4** *Bsp*HI, **5** *Pst*I, **6** *Sal*I, **7** *Nsp*I, **8** *Not*I, **9** *Xho*I. For details on enzymatic digestion reactions, see Table S7.

Supplementary Table 1: Genes sequenced in this study and their NCBI accession numbers.

Species	Gene	NCBI AN
Glyphotaelius pellucidus	U1 snRNA	MW149037
Hepialus humuli	U1 snRNA	MW149038
Tischeria ekebladella	U1 snRNA	MW149039
Tineola bisselliella	U1 snRNA	MW149040
Taleporia tubulosa	U1 snRNA	MW149041
Cameraria ohridella	U1 snRNA	MW149042
Yponomeuta evonymella	U1 snRNA	MW149043
Cydia pomonella	U1 snRNA	MW149044
Ephestia kuehniella	U1 snRNA	MW149045
Bombyx mori	U1 snRNA	MW149046
Glyphotaelius pelucidus	U2 snRNA	MW194851
Hepialus humuli	U2 snRNA	MW194852
Tischeria ekebladella	U2 snRNA	MW194853
Tineola bisselliella	U2 snRNA	MW194854
Taleporia tubulosa	U2 snRNA	MW194855
Cameraria ohridella	U2 snRNA	MW194856
Yponomeuta evonymella	U2 snRNA	MW194857
Cydia pomonella	U2 snRNA	MW194858
Ephestia kuehniella	U2 snRNA	MW194859
Bombyx mori	U2 snRNA	MW194860
Glyphotaelius pellucidus	5S rDNA	MW194861
Hepialus humuli	5S rDNA	MW194862
Tischeria ekebladella	5S rDNA	MW194863
Tineola bisselliella	5S rDNA	MW194864
Taleporia tubulosa	5S rDNA	MW194865
Cameraria ohridella	5S rDNA	MW194866
Yponomeuta evonymella	5S rDNA	MW194867
Cydia pomonella	5S rDNA	MW194868
Ephestia kuehniella	5S rDNA	MW194869
Bombyx mori	5S rDNA	MW194870
Phalera bucephala	histone H3	MW558903
Aglais urticae	histone H3	MW558904
Cossus cossus	histone H3	MW558905
Yponomeuta evonymella	histone H3	MW558906
Depressaria daucella	histone H3	MW558907
Spodoptera frugiperda	histone H3	MW558908
Tuta absoluta	histone H3	MW558909
Plutella xylostella	histone H3	MW558910
Abraxas grossulariata	histone H3	MW558911
Euthrix potatoria	histone H3	MW558912
Biston betularia	histone H3	MW558913
Tischeria ekebladella	histone H3	MW558914
Glyphotaelius pellucidus	histone H3	MW558915
Hepialus humuli	histone H3	MW558916
Pieris rapae	histone H3	MW558917
Cameraria ohridella	histone H3	MW558918
Hyalophora cecropia	histone H3	MW558919
Inachis io	histone H3	MW558920

Ephestia kuehniella	histone H3	MW558921
Gonepteryx rhamni	histone H3	MW558922
Tineola bisselliella	histone H3	MW558923
Limnaecia phragmitella	histone H3	MW558924
Cerura vinula	histone H3	MW558925
Taleporia tubulosa	histone H3	MW558926
Cydia pomonella	histone H3	MW558927
Proutia betulina	histone H3	MW558928
Bombyx mori	histone H3	MW558929

Supplementary table 2: Overview of results from this study and from literature.

Species	Classification	18S rDNA	Histone H3	5S rRNA	2n (F/M)	Karyotype – ref. [d]	18S rDNA – ref. [d]	Histone H3 – ref. [d]
Glyphotaelius pellucidus	Trichoptera, Limnephilidae	1 terminal	1 terminal on 3 bivalents	interstitial	59/60	[1]	this study	this study
Phymatopus californicus	Hepialidae	2 terminal	1 interstitial	not analysed	unknown		this study	this study
Hepialus humuli	Hepialidae	1 terminal	1 interstitial	interstitial	unknown		this study	this study
Tischeria ekebladella	Tischeridae	1 terminal	1 terminal	interstitial	46	[2]	this study	this study
Taleporia tubulosa	Psychidae	3 interstitial on 1 bivalent	1 interstitial	interstitial	59/60	[3]	this study	this study
Proutia betulina	Psychidae	2 terminal on 2 bivalents	1 interstitial	not analysed	61/62	[4]	this study	this study
Psyche crassiorella	Psychidae	1 terminal on 4 bivalents	1 interstitial	not analysed	61/62	[5]	this study	this study
Tineola bisselliella	Tineidae	1 terminal	1 interstitial	not detected	59/60	[6]	this study	this study
Cameraria ohridella	Gracillariidae	1 terminal	1 interstitial	not detected	60	[7]	this study	this study
Plutela xylostella	Plutellidae	1 terminal	1 terminal	not analysed	62	[8]	this study	this study
Yponomeuta evonymella	Yponomeutidae	1 terminal on 2 bivalents	1 terminal	not detected	61/62	[9]	this study	this study
Cydia pomonella	Tortricidae	2 terminal on 1 bivalent	1 interstitial	not detected	56	[10,11]	[11], this study	[12], this study
Zeiraphera griseana (Z. diniana)	Tortricidae	1 interstitial	not analysed	not analysed	56	Marec unpublished	Marec unpublished	
Grapholita molesta	Tortricidae	1 terminal	1 interstitial	not analysed	56	[12]	[12]	[12]
Grapholita funebrana	Tortricidae	1 interstitial	1 interstitial	not analysed	56	[12]	[12]	[12]
Lobesia botrana	Tortricidae	1 interstitial	1 interstitial	not analysed	56	[12]	[12]	[12]
Eupoecilia ambiguella	Tortricidae	1 terminal on 1 bivalent (ZW)	1 interstitial	not analysed	60	[12]	[12]	[12]
Cossus cossus	Cossidae	1 interstitial	1 terminal	not analysed	60 [a]	this study	this study	this study
Leptidea amurensis	Pieridae	1 terminal	1 terminal, 1 interstitial	not analysed	2n=118-122 [b]	[13]	[13]	[13]
Leptidea juvernica	Pieridae	1 interstitial	3-4 variable loci	not analysed	2n=80-91 [b]	[14]	[14]	[14]
Leptidea reali	Pieridae	1 terminal	1 interstitial	not analysed	2n=51-55 [b]	[14]	[14]	[14]
Leptidea sinapis	Pieridae	2 loci - variable positions	1-2 variable loci	not analysed	2n=56-106 [b]	[14]	[14]	[14]
Pieris brassicae	Pieridae	1 terminal	1 terminal	not analysed	30	[15]	[16], this study	this study

Pieris rapae	Pieridae	1 terminal	1 terminal	not analysed	50	[15]	[16], this study	this study
Gonepteryx rhamni	Pieridae	1 terminal	1 terminal	not analysed	62	[15]	this study	this study
Colias hyale	Pieridae	1 interstitial on 4 bivalents	not analysed	not analysed	62	[16]	[16]	
Polyommatus icarus	Lycaenidae	1 interstitial	not analysed	not analysed	46	[16]	[16]	
Inachis io	Nymphalidae	2 terminal on 4 bivalents, 1 terminal on 3 bivalents	1 interstitial	not analysed	62	[15]	[16], this study	this study
Aglais urticae	Nymphalidae	2 terminal on 1 bivalent, 1 terminal on 5 bivalents	1 interstitial	not analysed	62	[15]	this study	this study
Nymphalis xanthomelas	Nymphalidae	1 terminal on two bivalents	not analysed	not analysed	62	[16]	[16]	
Bicyclus anynana	Nymphalidae	1 terminal + 1 NOR, unknown position	not analysed	not analysed	56	[17]	[17]	
Depressaria daucella	Gelechiidae	1 interstitial	1 interstitial	not analysed	60	[18]	this study	this study
Limnecia phragmitella	Gelechiidae	1 terminal	1 interstitial	not analysed	unknown		this study	this study
Tuta absoluta	Gelechiidae	1 terminal on 2 bivalents	2 terminal on 1 bivalent	not analysed	58	[19]	this study	this study
Pectinop gossypiella	Gelechiidae	1 interstitial	not analysed	not analysed	60	[20]	[20]	
Ectomyelois ceratoniae	Pyralidae	1 terminal, 1 interstitial	not analysed	not analysed	62	[21]	[21]	
Ostrinia nubilalis	Pyralidae	1 terminal on 5 bivalents	not analysed	not analysed	62	[16]	[16]	
Ephestia kuehniella	Pyralidae	1 terminal on 2 bivalents	1 interstitial	not detected	60	[22]	[16,23]	this study
Cerura vinula	Notodontidae	1 terminal on 2 bivalents	1 interstitial	not analysed	42	[15]	this study	this study
Phalera bucephala	Notodontidae	1 terminal on 2 bivalents	1 interstitial	not analysed	60	[15]	this study	this study
Spodoptera frugiperda	Noctuidae	1 interstitial (autosome 11)	1 interstitial	not analysed	62	this study	this study	this study
Mamestra brassicae	Noctuidae	1 interstitial	not analysed	not analysed	62	[16]	[16]	
Phragmatobia fuliginosa	Erebidae	1 interstitial	not analysed	not analysed	58	[24]	[24]	
Arctia caja	Erebidae	1 interstitial	not analysed	not analysed	62	[16]	[16]	
Lymantria dispar	Erebidae	1 interstitial	not analysed	not analysed	62	[25]	[16]	
Orgyia recens	Erebidae	1 interstitial	not analysed	not analysed	60	[16]	[16]	

Orgyia leucostigma	Erebidae	1 interstitial	not analysed	not analysed	56	[16]	[16]	
Orgyia antiqua	Erebidae	1 interstitial	not analysed	not analysed	28	[26]	[16]	
Orgyia thyellina	Erebidae	1 interstitial	not analysed	not analysed	22	[27]	[27]	
Biston betularia	Geometridae	1 terminal on 3 bivalents	1 interstitial	not analysed	62	[28]	this study	this study
Abraxas grossulariata	Geometridae	1 terminal on 1 bivalent (ZW)	1 interstitial	not analysed	56	[29]	[29]	this study
Abraxas sylvata	Geometridae	1 terminal on 1 bivalent (ZW)	not analysed	not analysed	58	[29]	[29]	
Euthrix potatoria	Bombycidae	2 interstitial on 1 bivalent	1 interstitial	not analysed	62	[15]	this study	this study
Bombyx mori	Bombycidae	1 interstitial (autosome 11)	1 interstitial	not detected	56	[15]	[30]	this study
Manduca sexta	Sphingidae	1 interstitial	not analysed	not analysed	56	[31]	[16]	
Antheraea yamamai	Saturniidae	1 terminal on 2 bivalents	not analysed	not analysed	62	[32]	[32]	
Antheraea pernyi	Saturniidae	1 terminal	not analysed	not analysed	98	[16]	[16]	
Samia cynthia ssp.	Saturniidae	1 interstitial	not analysed	not analysed	25-28 [c]	[32]	[32]	
Hyalophora cecropia	Saturniidae	1 terminal on 3 bivalents	2 terminal on 1 bivalent, 1 terminal on 1 bivalent	not analysed	62	[33]	this study	this study

[a] preliminary diploid chromosome number determined only in male, female unknown

[b] variable

[c] chromosome numbers differ in subspecies (2n = 25/26 in S. c. sp. indet.; 2n = 26 in S. c. walkeri; 2n = 27/28 in S. c. ricini)

[d] 1. Kiauta, B. & Lankhorst, L. The chromosomes of the caddis-fly, *Glyphotaelius pellucidus* (Retzius, 1783) (Trichoptera: Limnephilidae, limnephilinae). *Genetica* **40**, 1–6 (1969).
2. Lukhtanov, V. A. Sex chromatin and sex chromosome systems in nonditrysian Lepidoptera (Insecta). *J. Zool. Syst. Evol. Res.* **38**, 73–79 (2000).
3. Seiler, J. Research on the sex-chromosomes of Psychidae (Lepidoptera). *Biol. Bull.* **36**, 399–404 (1919).
4. Hejníčková, M. *et al.* Absence of W chromosome in Psychidae moths and implications for the theory of sex chromosome evolution in Lepidoptera. *Genes* **10**, 1016; **10.3390/genes10121016** (2019).
5. Seiler, J. Geschlechtschromosomen-Untersuchungen an Psychiden. *Z. Indukt. Abstamm. Vererbungsl.* **31**, 1–99 (1922).
6. Dalíková, M. *et al.* New insights into the evolution of the W chromosome in Lepidoptera. *J. Hered.* **108**, 709–719 (2017).
7. Prins, J. D., Prins, W. D. & Dall'Asta, U. The karyotype of *Cameraria ohridella* (Lepidoptera: Gracillariidae). *Phegea* **301**, 5–10 (2002).
8. Yasukochi, Y. *et al.* Extensive conserved synteny of genes between the karyotypes of *Manduca sexta* and *Bombyx mori* revealed by BAC-FISH mapping. *PLoS ONE* **4**, e7465; 10.1371/journal.pone.0007465 (2009).

9. Nilsson, N.-O., 'Löfstedt, C. & Dävring, L. Unusual sex chromosome inheritance in six species of small ermine moths (Yponomeuta, Yponomeutidae, Lepidoptera). *Hereditas* **108**, 259–265 (1988).
10. Ortiz, E. & Templado, J. Los cromosomas de tres especies de tortrícidos (Lep. Tortricidae). *EOS-Rev. Esp. Entomol.* **51**, 77–84 (1976).
11. Fuková, I., Nguyen, P. & Marec, F. Codling moth cytogenetics: karyotype, chromosomal location of rDNA, and molecular differentiation of sex chromosomes. *Genome* **48**, 1083–1092 (2005).
12. Šíchová, J., Nguyen, P., Dalíková, M. & Marec, F. Chromosomal evolution in tortricid moths: Conserved karyotypes with diverged features. *PLoS ONE* **8**, e64520; 10.1371/journal.pone.0064520 (2013).
13. Šíchová, J. *et al.* Fissions, fusions, and translocations shaped the karyotype and multiple sex chromosome constitution of the northeast-Asian wood white butterfly, *Leptidea amurensis*. *Biol. J. Linn. Soc.* **118**, 457–471 (2016).
14. Šíchová, J. *et al.* Dynamic karyotype evolution and unique sex determination systems in Leptidea wood white butterflies. *BMC Evol Biol* **15**, 89; 10.1186/s12862-015-0375-4 (2015).
15. Robinson, R. *Lepidoptera Genetics*. (Pergamon Press, 1971).
16. Nguyen, P., Sahara, K., Yoshido, A. & Marec, F. Evolutionary dynamics of rDNA clusters on chromosomes of moths and butterflies (Lepidoptera). *Genetica* **138**, 343–354 (2010).
17. Van't Hof, A. E., Marec, F., Saccheri, I. J., Brakefield, P. M. & Zwaan, B. J. Cytogenetic characterization and AFLP-based genetic linkage mapping for the butterfly *Bicyclus anynana*, covering all 28 karyotyped chromosomes. *PLoS ONE* **3**, e3882; 10.1371/journal.pone.0003882 (2008).
18. Carabajal Paladino, L. Z. *et al.* Sex chromosome turnover in moths of the diverse superfamily Gelechioidea. *Genome Biol. Evol.* **11**, 1307–1319 (2019).
19. Carabajal Paladino, L. Z. *et al.* The effect of X-rays on cytological traits of *Tuta absoluta* (Lepidoptera: Gelechiidae). *Fla. Entomol.* **99**, 43–53 (2016).
20. Bartlett, A. & Del Fosse, F. The pachytene karyotype of the pink bollworm (Lepidoptera: Gelechiidae). *Southwest Entomol.* **16**, 223–235 (1991).
21. Mediouni, J., Fuková, I., Frydrychová, R., Dhouibi, M. H. & Marec, F. Karyotype, sex chromatin and sex chromosome differentiation in the carob moth, *Ectomyelois ceratoniae* (Lepidoptera: Pyralidae). *Caryologia* **57**, 184–194 (2004).
22. Schulz, H.-J. & Traut, W. The pachytene complement of the wildtype and a chromosome mutant strain of the flour moth, *Ephestia kuehniella* (Lepidoptera). *Genetica* **50**, 61–66 (1979).
23. Marec, F. & Traut, W. Synaptonemal complexes in female and male meiotic prophase of *Ephestia kuehniella* (Lepidoptera). *Heredity* **71**, 394–404 (1993).
24. Traut, W. & Marec, F. Sex chromosome differentiation in some species of Lepidoptera (Insecta). *Chromosome Res.* **5**, 283–291 (1997).
25. Krider, H. & Shields, K. Meiosis in North American and Asian races of gypsy moth (Lepidoptera: Lymantriidae) and their hybrids. *Ann. Entomol. Soc. Am.* **90**, 223–229 (1997).
26. Traut, W. & Clarke, C. A. Karyotype evolution by chromosome fusion in the moth *Genus Orgyia*. *Hereditas* **126**, 77–84 (1997).
27. Traut, W. & Clarke, C. A. Cytogenetics of a moth species with a low chromosome number, *Orgyia Thyellina*. *Hereditas* **125**, 277–283 (1996).
28. Van't Hof, A. E. *et al.* Linkage map of the peppered moth, *Biston betularia* (Lepidoptera, Geometridae): a model of industrial melanism. *Heredity* **110**, 283–295 (2013).
29. Zrzavá, M. *et al.* Sex chromosomes of the iconic moth *Abraxas grossulariata* (Lepidoptera, Geometridae) and its congener *A. sylvata*. *Genes* **9**, 279; **10.3390/genes9060279** (2018).
30. Yoshido, A., Bando, H., Yasukochi, Y. & Sahara, K. The *Bombyx mori* karyotype and the assignment of linkage groups. *Genetics* **170**, 675–685 (2005).

31. Sahara, K. *et al.* Conserved synteny of genes between chromosome 15 of *Bombyx mori* and a chromosome of *Manduca sexta* shown by five-color BAC-FISH. *Genome* **50**, 1061–1065 (2007).
32. Yoshido, A., Marec, F. & Sahara, K. Resolution of sex chromosome constitution by genomic in situ hybridization and fluorescence in situ hybridization with (TTAGG)n telomeric probe in some species of Lepidoptera. *Chromosoma* **114**, 193–202 (2005).
33. Bytinski-Salz, H. Untersuchungen an Lepidopteren-hybriden V. Die Verwandfschaft der Platysamia Arten (Lepidoptera, Saturniidae) nach untersuchungen iiber die Fertilitat und die chromosomen Verhatnisse ihrer Bastarde. *Arch. Exp. Zellforsch* 217–237 (1938).

Supplementary Table 3: Overview of the results from qPCR experiments.

Species	Target gene	Target gene to reference ratio (*R*) sample I	sample II	sample III	$E_{\mathrm{Referemce}}$	E_{Target}	mean	± S.D.
Glyphotaellius pellucidus	*5S rRNA*	53.322	61.585	46.555	0.82	0.813	53.821	7.527
	U1 snRNA	5.301	4.421	5.101	0.82	0.79	4.941	0.462
	U2 snRNA	7.835	8.025	6.683	0.82	0.759	7.514	0.726
Hepialus humuli	*5S rRNA*	232.102	317.306	242.304	0.937	0.985	263.904	46.528
	U1 snRNA	20.641	24.78	23.32	0.769	0.83	22.914	2.099
	U2 snRNA	19.577	15.064	15.693	0.769	0.84	16.778	2.444
Tischeria ekebladella	*5S rRNA*	18.445	18.81	22.522	0.822	0.891	19.926	2.255
	U1 snRNA	3.138	1.691	1.539	0.822	0.863	2.123	0.884
	U2 snRNA	3.246	2.998	3.121	0.822	0.817	3.122	0.124
Tineolla bisselliella	*5S rRNA*	12.917	14.8	16.227	0.978	0.972	14.648	1.661
	U1 snRNA	2.336	2.653	2.279	0.978	1.006	2.423	0.202
	U2 snRNA	0.509	0.899	1.57	0.978	0.927	0.993	0.537
Taleporia tubulosa	*5S rRNA*	50.283	54.301	49.178	1.01	1.02	51.254	2.696
	U1 snRNA	10.512	11.272	11.654	1.01	0.992	11.146	0.581
	U2 snRNA	54.614	45.474	67.531	1.01	1.09	55.873	11.083
Cameraria ohridella	*5S rRNA*	40.296	35.272	39.842	0.55	0.759	38.469	2.779
	U1 snRNA	4.088	4.135	4.365	0.855	0.886	4.196	0.148
	U2 snRNA	8.946	8.772	10.179	0.855	0.684	9.299	0.768
Yponomeuta evonymella	*5S rRNA*	6.121	5.875	6.012	0.89	0.971	6.003	0.123
	U1 snRNA	3.205	2.686	3.023	0.89	0.971	2.971	0.263
	U2 snRNA	3.566	3.171	3.613	0.89	0.928	3.449	0.243
Cydia pomonella	*5S rRNA*	9.932	14.56	15.362	0.903	0.928	13.285	2.931
	U1 snRNA	8.446	6.088	6.553	0.903	0.95	7.029	1.249
	U2 snRNA	21.848	20.913	21.931	0.903	0.843	21.564	0.565
Ephestia kuehniella	*5S rRNA*	17.019	17.518	16.534	0.984	1.046	17.024	0.491
	U1 snRNA	9.818	9.229	10.129	0.984	0.932	9.726	0.457
	U2 snRNA	9.409	9.833	10.527	1.006	0.935	9.923	0.565
Bombyx mori	*5S rRNA*	128.698	127.215	95.408	1.025	1.027	117.107	18.806
	U1 snRNA	27.288	33.625	34.646	1.001	0.889	31.853	3.986
	U2 snRNA	10.781	13.184	2.158	1.001	1.002	12.041	1.206

Supplementary table S4: List of primers used for PCR amplification of marker genes.

Gene	Forward primer	Reverse primer	Product length [bp]	Annealing temp. [°C]	Reference [a]
18 S rDNA	CGATACCGCGAATGGCTCAA	ACAAAGGGCAGGGACG	1850	58	[1]
Histone H3	ATGGCNCGTACNAARCARAC	TANGCACGYTCNCCNCGGAT	300–500	62	[2]
5S rDNA	GCCAACGTCCATACCAYGYTGA	AAGCCAACGNCACGYGGT	120	58	This study
U1 snRNA	CAAAAATTACGCGTCCGAG	CGTAGGGGACACCGTGAT	120	58	This study
U2 snRNA	AGATCAAAGTGTAGTATCTGTTC	CTGCAATGCCGGGCCRAC	140	58	This study

[a] 1. Fuková, I., Nguyen, P. & Marec, F. Codling moth cytogenetics: karyotype, chromosomal location of rDNA, and molecular differentiation of sex chromosomes. *Genome* **48**, 1083–1092; (2005).

2. Šíchová, J., Nguyen, P., Dalíková, M. & Marec, F. Chromosomal evolution in tortricid moths: Conserved karyotypes with diverged features. *PLoS One* **8**, e64520; 10.1371/journal.pone.0064520 (2013).

Supplementary table S5: List of species examined.

Superfamily	Species	Origin	Rearing conditions, diet
Limnephiloidea (Trichoptera)	*Glyphotaelius pellucidus*	Znojmo, CZ	fresh water, 15°C, *Fagus* sp.
Hepialoidea	*Phymatopus californicus*	Bodega Marine Reserve, California, USA	15°C, *Daucus carota*
	Hepialus humuli	Bochov, Czech Republic	outdoor container with planted *Daucus carota*
Tischerioidea	*Tischeria ekebladella* [a]	České Budějovice, CZ	ambient conditions, *Quercus* sp.
Tineoidea	*Taleporia tubulosa* [a]	České Budějovice, CZ	ambient conditions
	Proutia betulina [a]	České Budějovice, CZ	ambient conditions
	Psyche crassiorella [a]	České Budějovice, CZ	ambient conditions
	Tineola bisselliella	České Budějovice, CZ	25 ± 1°C, 16/8 h (light/dark) regime, wool
Gracillarioidea	*Cameraria ohridella* [a]	České Budějovice, CZ	ambient conditions, *Aesculus hippocastanum*
Yponomeutoidea	*Plutella xylostella*	Laboratory strain	25 ± 1°C, 16/8 h (light/dark) regime, artificial diet [1]
	Yponomeuta evonymella	Amsterdam, NL	ambient conditions, *Prunus padus*
Tortricoidea	*Cydia pomonella*	Laboratory strain Krym-61	27±1 °C ; 16/8 h (light/dark) regime, artificial diet [2]
Cossoidea	*Cossus cossus* [a]	České Budějovice, CZ	ambient conditions
Papilionoidea	*Pieris brassicae*	Levín, CZ	ambient conditions, *Brassica napus*
	Pieris rapae	Levín, CZ	ambient conditions, *Brassica oleracea*
	Gonepteryx rhamni [a]	Levín and České Budějovice, CZ	ambient conditions
	Inachis io	Vrábče, CZ	ambient conditions, *Urtica dioica*
	Aglais urticae	Vrábče, CZ	ambient conditions, *Urtica dioica*
Gelechioidea	*Depressaria daucella* [a]	Tábor, CZ	ambient conditions, *Carum* sp.
	Limnaecia phragmitella [a]	České Budějovice, CZ	ambient conditions, *Typha* sp.
	Tuta absoluta	Laboratory strain	25 ± 1°C, 16/8 h (light/dark) regime, *Solanum lycopersicum* [3]
Pyraloidea	*Ephestia kuehniella*	Laboratory strain WT-C02	25 ± 1°C, 16/8 h (light/dark) regime, artificial diet [4]
Noctuoidea	*Cerura vinula*	Chrudim, CZ	ambient conditions, *Salix alba*
	Phalera bucephala [a]	České Budějovice, CZ	
	Spodoptera frugiperda	Laboratory of Heckel D, Jena, DE	25 ± 1°C, 16/8 h (light/dark) regime, artificial diet [5]
Geometroidea	*Biston betularia*	Laboratory of Saccheri I, Liverpool,UK	ambient conditions, *Betula pendula* and *Malus domestica*
	Abraxas grossulariata	Near Hrabětice, CZ	Ambient conditions, *Ribes rubrum* [6]
Bombycoidea	*Euthrix potatoria*	Sedlec, CZ	ambient conditions, *Poaceae*
	Bombyx mori	Laboratory strain p50	ambient conditions, *Morus* sp.
	Hyalophora cecropia	Connecticut, USA	ambient conditions, *Acer platanoides*

[a] Individuals processed immediately after collection

1. details in Shelton, A.M. *et al.* Comparative analysis of two rearing procedures for diamondback moth (Lepidoptera: Plutellidae). *J. Entomol. Sci.* **26**, 17–26 (1991).

2. details in Fuková. I., Nguyen, P. & Marec, F. Codling moth cytogenetics: karyotype, chromosomal location of rDNA, and molecular differentiation of sex chromosomes. *Genome* **48**, 1083–1092 (2005).

3. Cagnotti, C.L. *et al.* Effects of X-rays on *Tuta absoluta* for use in inherited sterility programmes. *J. Pest Sci.* **85**, 413–421 (2012).

4. Marec, F. Genetic control of pest Lepidoptera: induction of sex-linked recessive lethal mutations in *Ephestia kuehniella* (Pyralidae). *Acta Entomol. Bohemoslov.* **87**, 445—458(1990)

5. Kost, S. *et al.* A Z-linked sterility locus causes sexual abstinence in hybrid females and facilitates speciation in *Spodoptera frugiperda*. *Evolution* **70**, 1418–1427 (2016).

6. Zrzavá, M. *et al.* Sex chromosomes of the iconic moth *Abraxas grossulariata* (Lepidoptera, Geometridae) and its congener *A. sylvata*. *Genes* **9**, 279; 10.3390/genes9060279 (2018).

Supplementary Table 6: Primers and their concentrations used in the qPCR experiments.

Species	Gene	Forward	Reverse	Reference [a]	Final conc.
Glyphotaelius pelucidus	*Acetylcholinesterase 2*	CCCCGGATTCGAAGGAGAAG	ATCCGCCGCCATAAATCCAA	this study	0,8 µM
	U1 snRNA	TAGGGAAAATCGCAAGGGTCAG	GTAGGGGACACCGTGATCAAT	this study	0,8 µM
	U2 snRNA	GTGGAGCAAGCCCCGAACATC	GCAATGCGGGACCAGTATATT	this study	0,8 µM
	5S rDNA	GCCAACGTCCATACCATGCTGA	ACCGCGTGTCGTTGGCTT	this study	0,8 µM
Hepialus humuli	*paralytic*	ATTCCCTTGGCTCCCTTAAC	CCCACATTGCTGAGAGTGG	[1]	1,2 µM
	U1 snRNA	TAGGGAAAATCGCAAGGGTCAG	CCGTGATCATGAAGGCGGTA	this study	0,8 µM
	U2 snRNA	GTGGAGCAAGCCCCGAACATC	ATCTGATAGTTCCCGCATTGC	this study	0,8 µM
	5S rDNA	TGAAAACACCGGTTCTCGTCC	CACCCATCCAAGTACTGACCG	this study	0,4 µM
Tischeria ekebladella	*Acetylcholinesterase 2*	GACACTATTGTGGCCTCGATG	CCAGGAGTCTCTTCACTTTGC	[2]	0,8 µM
	U1 snRNA	ATTACGCGTCCGAGATATCCA	TTGCACTAGGGTTGGGTTGAC	this study	0,8 µM
	U2 snRNA	AGCCCCTATCTCCGCATCAG	GCTTAATATCTGAAAGTTCCCGCAA	this study	0,8 µM
	5S rDNA	CCCATCCAAGTACTGACCACG	TGAATACACCGGTTCTCGTCC	this study	0,8 µM
Tineola bisselliella	*Acetylcholinesterase 2*	GAGGCACCCAAATATTCAAATACAA	ATACGAATATTTTCCAGGATTTGAGG	[2]	1 µM
	U1 snRNA	GGGTAATCACAGGGGTCAACC	ATCAAGAAGGCGGTTCCCC	this study	0,4 µM
	U2 snRNA	GTGGAGCAAGCCCCGAACATC	ATCTGATAGTTCCCGCATTGC	this study	0,8 µM
	5S rDNA	ACCCATCCAAGTACTGACCAC	TACACCGGTTCTCGTCCGAT	this study	0,8 µM
Taleporia tubulosa	*Acetylcholinesterase 2*	TGGCGGAAAGACCTAAGGTTC	TACAAGTCTAGGGTCGCAGTG	this study	0,4 µM
	U1 snRNA	CCCACATTAGGGGTAATCGCA	CGTGATCACAAAGGCGGTTC	this study	0,4 µM
	U2 snRNA	TCAGCTTAATATCTGAAAGTTCCAG	TGGAGCGAGCCCCGAACAT	this study	0,4 µM
	5S rDNA	GCGGTCACCCATCCAAGTAC	TGAATACACCGGTTCTCGTCC	this study	0,4 µM
Cameraria ohridella	*Acetylcholinesterase 2*	TACACGGACTGGGAGGAGATAAC	TAGTTTGTAGGGCAGATGAAGAAG	[2]	0,8 µM
	U1 snRNA	CCGTGATCAAGTAGGCGGTT	TAGGGATAATCGCAGGGGTCA	this study	0,8 µM
	U2 snRNA	CAGCTTAATATCTGAAAGTTCCCTCAC	AGCCCCAACTACCCATCTAGA	this study	0,8 µM
	5S rDNA	TGAAAACACCGGTTCTCGTCC	CACCCATCCAAGTACTGACCG	this study	0,8 µM
Yponomeuta evonymella	*Acetylcholinesterase 2*	AGCTGGAGCTGTTTCTGTCTC	CGCATAATGCTCTCTTCTCTTG	this study	0,8 µM
	U1 snRNA	CCGTGATCAAGTAGGCGGTT	TAGGGATAATCGCAGGGGTCA	this study	0,8 µM
	U2 snRNA	GTAACGGGAGTGGAGCGAG	CAGCTTAATATCTGAAAGTTCCCCC	this study	0,8 µM
	5S rDNA	AGCCAACGACACGTGGTG	TGAATACACCGGTTCTCGTCC	this study	0,8 µM
Cydia pomonella	*Acetylcholinesterase 2*	CTGCCACATTCATGCGTTCA	ACCCAAAGCATAACAGCTGC	this study	0,4 µM
	U1 snRNA	CAAAAATTACGCGTCCGAG	CGTAGGGGACACCGTGAT	this study	0,4 µM
	U2 snRNA	AGTGTAGTATCTGTTCTTTTCAGC	CCGTGACGGGAGTGGAG	this study	0,4 µM
	5S rDNA	CCAACGTCCATACCATGCTGAAT	TCACCCATCCAAGTACTGACCT	this study	0,4 µM

Ephestia kuehniella	*Acetylcholinesterase 2*	CCGGCATCATCAAGACAAACC	GTACCACTCATGTAGCCACCG	this study	0,4 µM
	U1 snRNA	TCCGAGTTATCCACATTAGGGA	GAAGGCGGTTCCCCCAGG	this study	0,4 µM
	U2 snRNA	GAGCGAGCCCCGAACATC	CAGCTTAATATCTGAAAGTTCCCCC	this study	0,8 µM
	5S rDNA	ACCCATCCAAGTACTGACCAC	TGAAAACACCGGTTCTCGTCC	this study	0,4 µM
Bombyx mori	*Acetylcholinesterase 2*	GCCTTTGGACGGGAATATTTA	CGTAGACCACGACCAACTGA	this study	0,4 µM
	U1 snRNA	TACTCCATTGCACTGCGGAG	AATTACGCGTCCGAGTTACCC	this study	0,4 µM
	U2 snRNA	CGGCCTTTTGGCTAAGATCA	GGAGCGAGCCCCTAACATC	this study	0,4 µM
	5S rDNA	TGAAAACACCGGTTCTCGTCC	CACCCATCCAAGTACTGACCG	this study	0,8 µM

[a] 1. Dalíková, M. *et al.* New Insights into the Evolution of the W Chromosome in Lepidoptera. *J. Hered.* **108**, 709–719(2017).

Voleníková, A. Karyotype and sex chromosomes analysis of two species from basal lepidopteran family Hepialidae (Lepidoptera: Hepialidae). Master thesis (in Czech), University of South Bohemia, České Budějovice, Czech Republic (2014).

Supplementary table S7: Information about digestion reactions used for Southern hybridization.

Species	Gene	Restriction enzymes I	Restriction enzymes II	Restriction enzymes III
Glyphotaelius pellucidus	*U1 snRNA*	*Afe*I + *Xba*I [a]	*Afe*I + *Bam*HI [a]	*Pst*I + *Sal*I [a]
	5S rDNA	*Afe*I + *Bam*HI [a]	*Pst*I + *Sal*I [a]	*Afe*I + *Xba*I [a]
Hepialus humuli	*U1 snRNA*	*Afe*I + *Xba*I [a]	*Afe*I + *Bam*HI [a]	*Pst*I + *Sal*I [a]
	5S rDNA	*Afe*I + *Xba*I [a]	*Afe*I + *Bam*HI [a]	*Pst*I + *Sal*I [a]
Tischeria ekebladella	*U1 snRNA*	-	-	-
	5S rDNA	*Pst*I + *Bsp*HI [a]	*Xba*I + *Bam*HI [a]	*Xba*I + *Nsp*I [a]
Taleporia tubulosa	*U1 snRNA*	-	-	-
	5S rDNA	*Afe*I + *Xba*I [a]	*Afe*I + *Bam*HI [a]	*Pst*I + *Sal*I [a]
Tineola bisselliella	*U1 snRNA*	*Afe*I + *Xba*I [a]	*Afe*I + *Bam*HI [a]	*Pst*I + *Sal*I [a]
	5S rDNA	*Afe*I + *Xba*I [a]	*Afe*I + *Bam*HI [a]	*Pst*I + *Sal*I [a]
Cameraria ohridella	*U1 snRNA*	*Afe*I + *Xba*I [a]	*Afe*I + *Bam*HI [a]	*Pst*I + *Sal*I [a]
	5S rDNA	*Afe*I + *Xba*I [a]	*Afe*I + *Bam*HI [a]	*Pst*I + *Sal*I [a]
Yponomeuta evonymella	*U1 snRNA*	*Not*I + *XhoI* [b]	*Afe*I + *Bam*HI [a]	*Bam*HI + *Sal*I [a]
	5S rDNA	*Pst*I + *Bsp*HI [a]	*Bsp*HI + *Bam*HI [a]	*Xba*I + *Nsp*I [a]
Ephestia kuehniella	*U1 snRNA*	*Pst*I + *Bsp*HI [a]	*Xba*I + *Bam*HI [a]	*Xba*I + *Nsp*I [a]
	5S rDNA	*Pst*I + *Bsp*HI [a]	*Xba*I + *Bam*HI [a]	*Xba*I + *Nsp*I [a]
Cydia pomonella	*U1 snRNA*	*Not*I + *Pst*I [b]	*Bam*HI + *Sal*I [a]	*Afe*I + *Bam*HI [a]
	5S rDNA	*Not*I + *Pst*I [b]	*Bam*HI + *Sal*I [a]	*Afe*I + *Bam*HI [a]
Bombyx mori	*U1 snRNA*	*Pst*I + *Bsp*HI [a]	*Xba*I + *Bam*HI [a]	*Xba*I + *Nsp*I [a]
	5S rDNA	*Pst*I + *Bsp*HI [a]	*Bsp*HI + *Bam*HI [a]	*Xba*I + *Nsp*I [a]

[a] CutSmart buffer, NEB (New England Biolabs, Ipswich, MA, USA)

[b] Buffer H + BSA + Triton (ThermoFisher Scientific, Waltham, MA, USA)
